# Dynamic SPECT imaging kinetic modelling characterises tracer influx and efflux in tumour-infiltrating CAR T cells

**DOI:** 10.1186/s13550-026-01492-y

**Published:** 2026-08-29

**Authors:** Eva Bugallo-Blanco, Jan Taprogge, Iain Murray, Julie Cleaver, Robert Page, Olivier Martinez, Jane Sosabowski, Sophie Papa

**Affiliations:** 1 Immunoengineering Group, King s College London, 3rd floor Bermondsey Wing, Great Maze Pond, London, SE1 9RT UK; 2 Cancer Imaging Laboratory, Centre for Cancer Biomarkers and Biotherapeutics, Barts Cancer Institute, Charterhouse Square, Queen Mary University of London, London, UK; 3 Present Address: Research Department of Haematology, University College London, London, UK; 4 Joint Department of Physics, Royal Marsden NHSFT, Sutton, UK; 5 Institute of Cancer Research, Sutton, UK

**Keywords:** Cell tracking, SPECT/CT, Adoptive cell transfer, Sodium iodide symporter, Kinetic modelling

## Abstract

**Background:**

Chimeric Antigen Receptor (CAR) T cell therapies for solid tumours face challenges regarding inefficient trafficking to the tumour site, limited extravasation and impaired persistence within an immunosuppressive microenvironment. Non-invasive imaging approaches capable of capturing the dynamic behaviour and biodistribution of CAR T cells are therefore needed to better understand these barriers. This study explores the use of compartmental modelling to interpret technetium-99 m pertechnetate ([^99m^Tc]TcO_4_^−^) uptake by CAR T cells co-expressing the sodium iodide symporter (NIS) reporter gene in a prostate cancer NSG mouse model using single-photon emission computed tomography computerised tomography (SPECT/CT).

**Results:**

Co-expression of hNIS with a prostate-specific membrane antigen (PSMA) targeting CAR did not impair CAR T cell function and enabled non-invasive visualisation of CAR-hNIS T cell infiltration *in vivo*. Dynamic SPECT/CT imaging revealed clear differences in tumour uptake between CAR-hNIS treated mice and control groups. Compartmental modelling using a reversible two-compartment framework demonstrated increased tracer influx and reduced tracer efflux in CAR-hNIS treated tumours. These kinetic changes indicate that tumour signal arises from NIS-mediated tracer uptake and delayed tracer washout in tumours containing infiltrating CAR T cells, rather than from passive perfusion or non-specific delivery alone. Immunohistochemistry confirmed CAR T cell infiltration in the tumours.

**Conclusions:**

This work establishes a proof-of-concept for applying kinetic modelling to interpret CAR T cell infiltration *in vivo* using dynamic hNIS-based SPECT imaging. By distinguishing tracer delivery from tracer washout kinetics and correcting for variability in tracer bioavailability, this approach provides a more sophisticated biological interpretation of tumour uptake than static imaging alone. These findings highlight the potential of integrating kinetic modelling into future quantitative cell-tracking strategies to improve the evaluation and optimisation of CAR T cell therapies for solid tumours.

**Supplementary Information:**

The online version contains supplementary material available at https://doi.org/10.1186/s13550-026-01492-y.

## Background

Chimeric Antigen Receptor (CAR) T cell therapy has demonstrated remarkable clinical success in haematological malignancies targeting CD19 antigen in B cells [1–4], yet comparable efficacy has not been achieved in solid tumours. While CAR T cell clinical trials targeting a variety of tumour-associated agents in solid tumours have reported outcomes from individual participants ranging from no efficacy to complete remission [5–8], the response to treatment remains inconsistent and generally inferior compared to haematological cancers. This disparity is attributed to multiple barriers presented by the solid tumour microenvironment (TME), including tumour heterogeneity, inefficient CAR T cell homing to the tumour site, the presence of immunosuppressive cells and cytokines, and nutrient and oxygen depletion within the tumour mass [9]. As CAR T cells are “living drugs”, their efficacy depends on dynamic process including trafficking, expansion and persistence, yet these processes are poorly understood.

Current monitoring methods following CAR T cell infusion, such as peripheral blood sampling and tumour biopsies, provide limited and static information and fail to capture whole-body biodistribution or temporal dynamics [10]. Consequently, improving our understanding of CAR T cell kinetics and biodistribution through non-invasive, quantitative imaging approaches that enable longitudinal monitoring of CAR T cells *in vivo* is crucial for optimising their therapeutic efficacy.

Molecular imaging techniques such as Single Photon Emission Computed Tomography (SPECT) and Positron Emission Tomography (PET) combined with computed tomography (CT) for anatomical reference offer non-invasive, three-dimensional, and longitudinal assessment of adoptively transferred immune cells [11–14], offering high sensitivity and translational potential [10, 15]. Direct and indirect labelling of CAR T cells has been used in preclinical studies. While direct cell labelling strategies enable early tracking, they are limited by rapid signal decay due to the short half-life of radionuclides and signal dilution following CAR T cell proliferation. Additionally, radiotracer efflux and phagocytosis of labelled dead cells can lead to inaccurate interpretation of their spatial distribution and inability to distinguish between viable and non-viable cells [16–18]. In contrast, reporter gene-based approaches provide a more reliable means for long-term monitoring of viable cells [19, 20].

The human sodium iodide symporter (hNIS) is an attractive candidate for non-invasive, longitudinal tracking of CAR T cells due to its human origin, limited endogenous expression, lack of internalisation upon interaction with the tracer [21] and its activity is restricted to viable cells with a functional Na^+^/K^+^ pump [22, 23], thereby enhancing sensitivity and specificity. It can transport clinically approved SPECT and PET tracers such as [^99m^Tc]TcO_4_^−^ or [^18^F]Tetrafluoroborate [22, 24, 25]. Preclinical studies have validated the use of hNIS-expressing CAR T cells through static PET and SPECT imaging [12, 26–28], yet the impact of dynamic physiological factors such as blood flow and radiotracer availability in circulation is often overlooked.

Dynamic imaging combined with kinetic modelling offers an opportunity to move beyond static uptake measurements by offering insight into the process governing tracer accumulation over time. Compartment modelling uses first-order differential equations to describe tracer exchange between blood and tissue compartments (Fig. S1), enabling estimation of parameters related to tracer delivery and retention [29–31].

In this study, we apply compartmental modelling to dynamic SPECT imaging to characterise the *in vivo* kinetics of [^99m^Tc]TcO_4_^−^ uptake by hNIS-expressing CAR-T cells in a prostate cancer mouse model. By integrating *in vitro* validation, dynamic *in vivo* imaging, and *ex vivo* confirmation of CAR T cell infiltration, we establish a proof-of-concept for using kinetic modelling to interpret tumour-associated tracer uptake while accounting for physiological factors such as tracer availability, vascularity delivery and reversible transport. This approach provides complementary information beyond standard tracer quantitation by revealing the physiological basis of tracer signal, distinguishing between increased tracer accumulation, prolonged tracer retention, or a combination of both.

## Results

### Generation and characterisation of hNIS-expressing CAR T cells for *in vivo* tracking

**Fig. 1:**
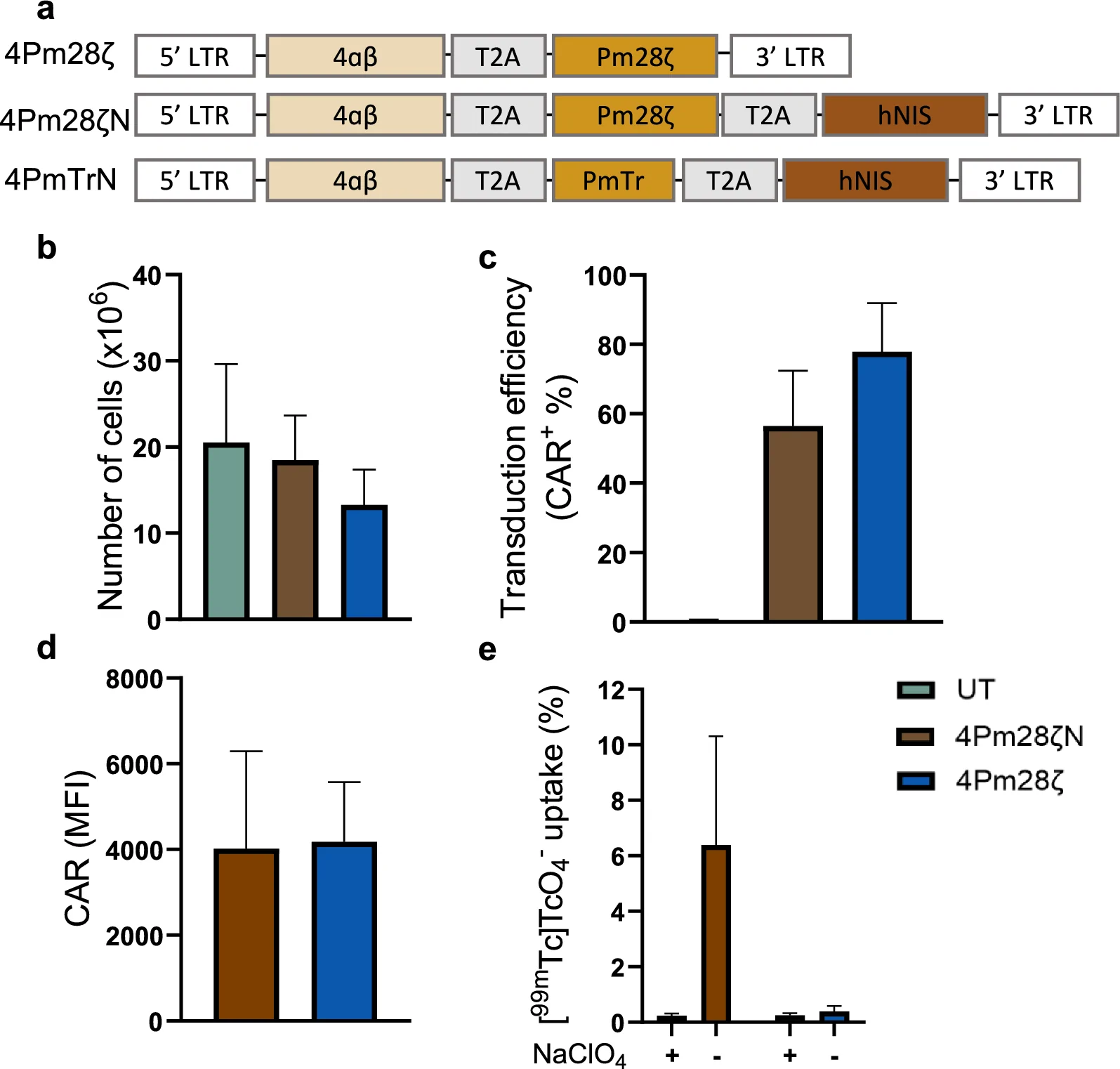
Impact of hNIS co-expression on CAR T cell generation. **A**) Schematic representation of PSMA-targeted CAR constructs. LTR: long terminal repeat. **B**) Total cell counts 11 days post transduction. **C**) Transduction efficiency measured by surface myc tag expression. **D**) Mean fluorescence intensity (MFI) of myc-tagged CAR-positive cells. **E**) *In vitro* [^99m^Tc]TcO_4_^−^ uptake in 4Pm28ζN and 4Pm28ζ CAR T cells in the presence or absence of an inhibitor at the end of the expansion period. n = 3 donors. UT = untransduced. Graphs represent mean ± SD

To assess the impact of hNIS co-expression on CAR T cell functionality, we engineered and characterised multiple CAR constructs. The 4Pm28ζN construct incorporates a PSMA-targeting scFv fused to a modified CD28 hinge containing a myc tag, CD28 transmembrane and costimulatory domains, aCD3ζ activation domain, and the reporter gene hNIS. A control CAR, 4Pm28ζ, which lacks hNIS was created for direct comparison of CAR functionality, while 4PmTrN construct was generated by truncating the intracellular domains, allowing target binding without activation in the presence of hNIS (Fig. 1a). The 4αβ chimeric cytokine receptor was included upstream of the CARs to enable selective expansion of transduced cells in interleukin-4 (IL-4) [32].

CAR T cell proliferation remained comparable across constructs following an 11-day expansion (Fig. 1b). Transduction efficiency, determined via myc tag detection by flow cytometry, was higher for 4Pm28ζ T cells than for 4Pm28ζN T cells (Fig. 1c), although surface expression levels, measured by median fluorescence intensity (MFI), were similar (Fig. 1d). Functional hNIS expression was confirmed through [^99m^Tc]TcO_4_^−^ uptake assays, with uptake observed in 4Pm28ζN T cells but not in control 4Pm28ζ T cells. The uptake was blocked in the presence of inhibitor sodium perchlorate (NaClO_4_) (Fig. 1e).

The functionality of 4Pm28ζN CAR T cells was assessed through cytotoxicity assays using the prostate cancer cell line PC3-LN3, engineered to overexpress PSMA (PLP) (Fig. S2a). Live-cell imaging demonstrated progressive tumour cell clearance by 4Pm28ζ and 4Pm28ζN CAR T cells in a dose-dependent manner. When co-cultured with the wild-type PC3-LN3 (PL) target cell line, cytotoxicity was detected only at the higher effector ratios after 50 h, consistent with undetectable PSMA levels by flow cytometry (Fig. S3a). Untransduced (UT) cells did not induce cytotoxicity when co-cultured with either tumour cell line (Fig. S3b). These data demonstrate the preservation of the potent anti-tumoral activity by 4Pm28ζN CAR T cells, irrespective of the expression of hNIS.

**Fig. 2:**
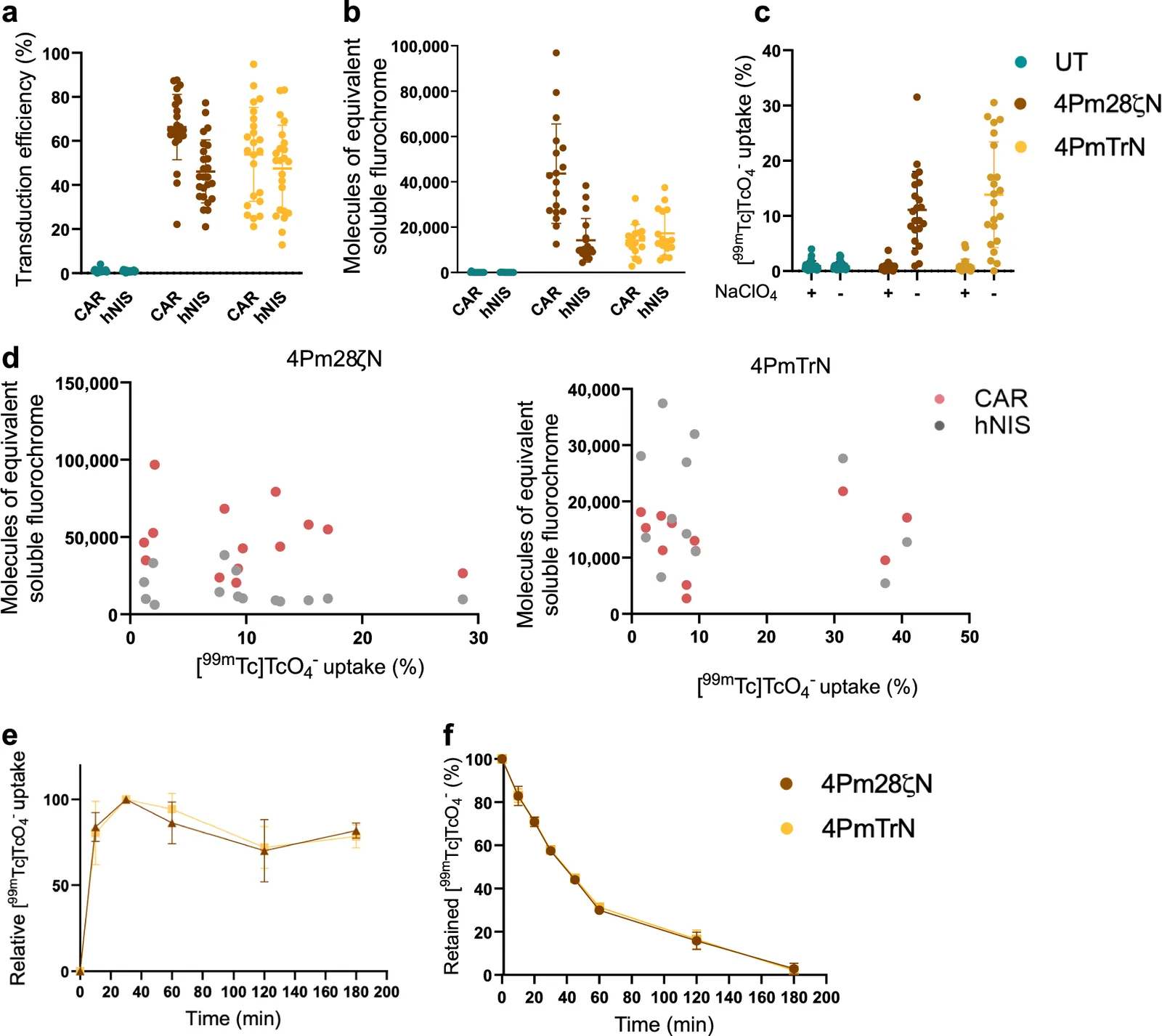
hNIS mediated [^99m^Tc]TcO_4_^−^ uptake in CAR T cells. **A**) Transduction efficiency at day 11, measuring CAR and hNIS surface expression levels. n = 15 donors/group. **B**) Quantification of CAR and hNIS surface molecules at day 11. n = 15 donors/group. **C**) *In vitro* [^99m^Tc]TcO_4_^−^ uptake in CAR T cells in the presence or absence of the inhibitor. n = 15 donors/group. **D**) Correlation between [^99m^Tc]TcO_4_^−^ uptake and quantified CAR and hNIS surface expression. n = 12 donors/group. **E**) Relative [^99m^Tc]TcO_4_^−^ uptake over three hours. n = 3 donors/group. **F**) Relative [^99m^Tc]TcO_4_^−^ efflux over three hours. n = 3 donors/group. Graphs represent mean ± SD of assays performed in triplicates

Flow cytometry analysis of hNIS expression in 4Pm28ζN and 4PmTrN revealed donor-dependent variability in transduction efficiency, with surface expression ranging from 20 to 80% (Fig. 2a). CAR surface density was higher in 4Pm28ζN T cells (44,822 ± 21,726 CARs/cell) than in 4PmTrN T cells (15,757 ± 6,557 CARs/cell), though greater variability was also observed in 4Pm28ζN T cells. In contrast, hNIS surface expression was slightly higher in 4PmTrN T cells (15,671 ± 9,632 hNIS/cell) compared to 4Pm28ζN T cells (11,644 ± 7,106 hNIS/cell) (Fig. 2b). Despite this variability, no correlation was observed between surface expression levels and *in vitro* [^99m^Tc]TcO_4_^−^ uptake, which also exhibited donor variability (Fig. 2c&d).

Radiotracer uptake kinetics revealed rapid influx of [^99m^Tc]TcO_4_^−^ with peak uptake at 30 min, retaining 70% of the maximal signal at 2 h post-exposure to the tracer (Fig. 2e). Decreasing the extracellular concentration of [^99m^Tc]TcO_4_^−^ to simulate *in vivo* conditions led to efflux of intracellular radionuclides with less than 50% of the radiotracer retained in the CAR T cells after 30 min (Fig. 2f).

### Radiolabelling CAR T cells with [^99m^Tc]TcO_4_^−^ preserves viability and functionality

Radiolabelling CAR T cells with [^99m^Tc]TcO_4_^−^ enables *in vivo* tracking but may also induce radiation-mediated DNA damage, potentially affecting functionality. To assess exposure levels, tracer kinetics and biodistribution were studied *in vivo.* Following intravenous administration, blood radioactivity peaked at 1 min, followed by an exponential decay phase to steady state by 60 min. Radiotracer concentrations ranged from about 1.2 MBq/ml at two minutes post-injection to 0.5 MBq/ml at steady state (Fig. S4a). *Ex vivo* biodistribution confirmed tracer uptake in untreated tumours at around 2% ID/g (0.2 MBq/ml) and physiological [^99m^Tc]TcO_4_^−^ uptake in the salivary glands and stomach (Fig. S4b).

**Fig. 3:**
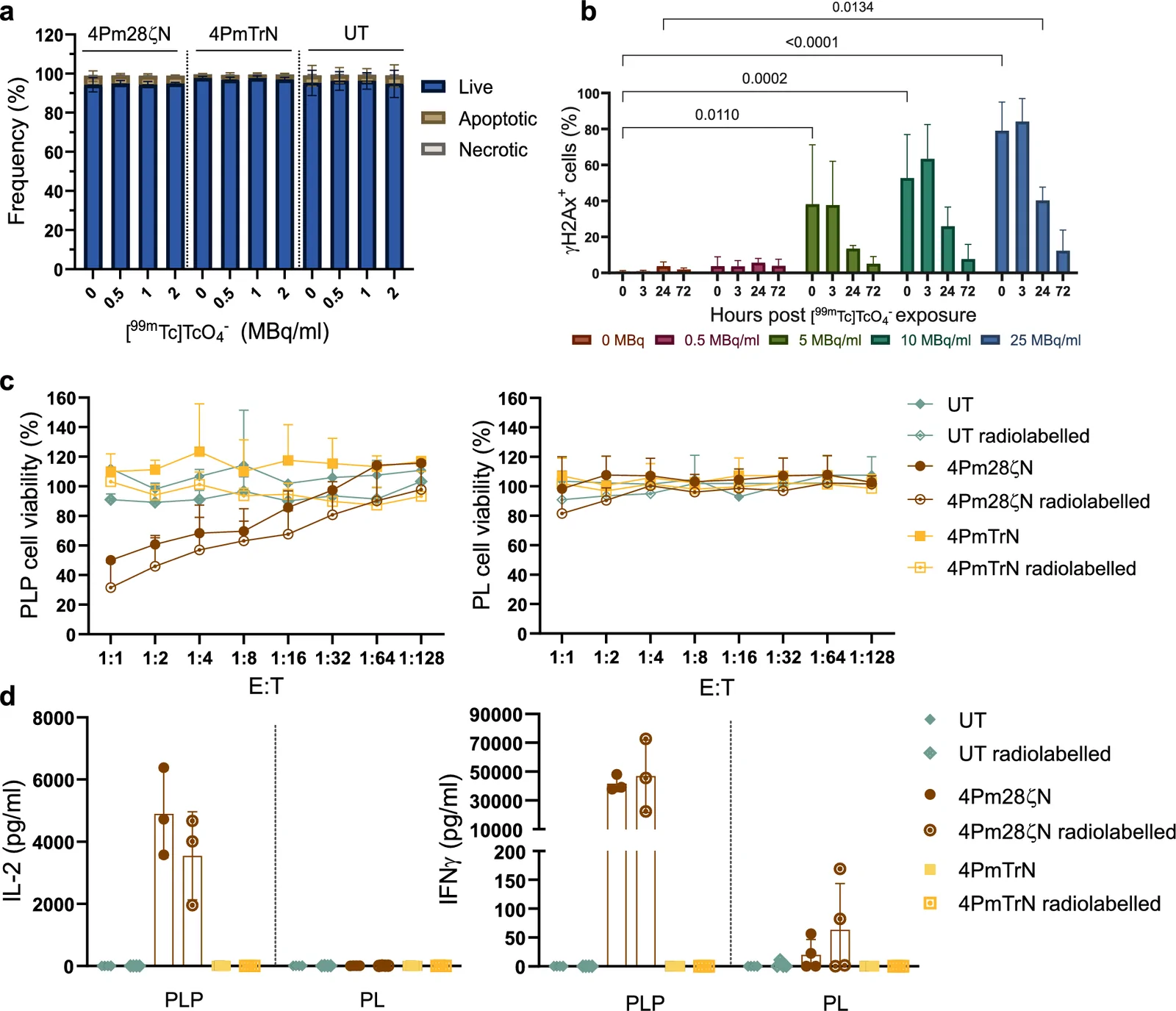
Exposure to tracer does not impact CAR T cell functionality. **A**) CAR T cell viability 24 h after exposure to tracer determined by Annexin V and Zombie near-infrared L/D dye staining. n = 3 donors/group. **B**) Percentage of 4Pm28ζN T cells expressing phosphorylated γH2Ax at 4 different timepoints after incubation with varying [^99m^Tc]TcO_4_^−^ concentrations. n = 3 donors/group. **C**) Tumour cell viability 48 h after co-culture with unlabelled or radiolabelled CAR T cells (1 MBq/ml) determined by MTT assays. n = 3–4/group. **D**) Cytokine production quantified from co-cultures in (C). n = 3–4/group. UT = untransduced T cells. Graphs represent mean ± SD. Statistical analysis performed by two-way ANOVA

*In vitro* exposure to perfusion tracer concentrations (0.2–0.5 MBq/ml) did not affect cell viability or induce DNA-damage in 4Pm28ζN, 4PmTrN or UT T cells (Fig. 3a ). At higher concentrations (> 5 MBq/ml), increased levels of apoptosis and necrosis were quantified in hNIS expressing cells (4Pm28ζN and 4PmTrN), but not in UT cells (data not shown). A dose-dependent increase in phosphorylated γH2Ax was observed in 4Pm28ζN T cells at > 5 MBq of [^99m^Tc]TcO_4_^−^, with DNA-damage repairing within 24 h, except at 25 MBq of [^99m^Tc]TcO_4_^−^, where recovery required 72 h (Fig. 3b).

Exposure of the CAR T cells to tracer did not impair their anti-tumoral activity (Fig. 3c) or cytokine production (Fig. 3d). Radiolabelled 4Pm28ζN T cells demonstrated robust antigen-specific tumour cell lysis and interleukin-2 (IL-2) and interferon-gamma (IFNγ) cytokine production when co-cultured with PLP target cells, with no cytotoxicity against non-PSMA expressing PL cell lines, comparable to unlabelled controls. 4PmTrN cells and UT T cells did not mount a cytotoxic response. These data demonstrate that radiotracer uptake does not compromise CAR T cell viability or function, meeting the key kinetic modelling principle of non-interference with the process being studied.

### CAR T cells demonstrate robust anti-tumoral activity *in vivo*

**Fig. 4:**
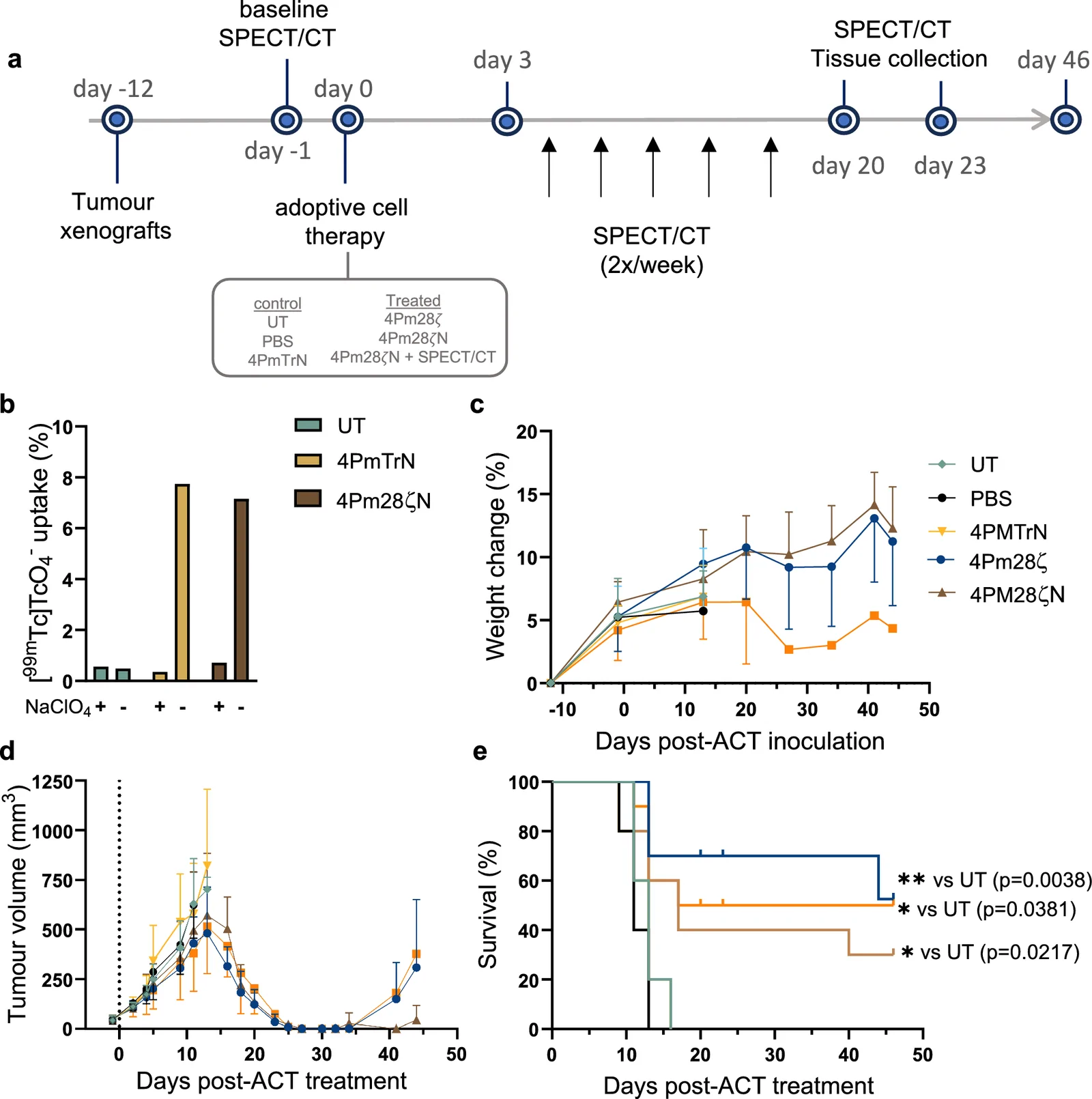
Assessing the cytotoxicity of 4Pm28ζN *in vivo.* **A**) Experimental timeline of tumour xenograft model, SPECT/CT imaging and *ex vivo* analysis. **B**) *In vitro* [^99m^Tc]TcO_4_^−^ uptake in administered CAR T cells in the presence or absence of the inhibitor. **C**) Percentage body weight change. Dashed line represents ACT day. **D**) Tumour volume over time using calliper measurements. **E**) Kaplan–Meier survival curve. Vertical marks indicate censored animals (n = 3) due to elective euthanasia for tissue collection following imaging sessions. n = 10 (treated groups), n = 5 (control groups). Statistical analysis performed by Log-rank (Mantel-Cox) test. ACT: adoptive cell transfer. Graphs represent mean ± SD

To evaluate the *in vivo* efficacy of hNIS-expressing CAR T cells, subcutaneous PLP xenograft tumours were established in mice, and tumour-bearing mice were treated with 4Pm28ζ, 4Pm28ζN or radiolabelled 4Pm28ζN T cells. Controls included PBS, as well as UT, and 4PmTrN CAR T cell groups. Established tumours were treated on day 12 when the average tumour volume was 46 mm^3^, and tumour growth was monitored by calliper measurements (Fig. 4a). On the day of adoptive cell transfer (ACT), flow cytometry analysis of CAR expression showed transduction efficiencies above 80% (Fig. S5). Radiotracer uptake assays confirmed [^99m^Tc]TcO_4_^−^ internalisation by 4Pm28ζN (7.7%) and 4PmTrN (7.1%) T cells (Fig. 4b). Animals were treated with two million CAR^+^ T cells or untransduced cells. All groups maintained stable body weights, though reduced body weight gain was observed in mice receiving repeated twice-weekly [^99m^Tc]TcO_4_^−^ administration (Fig. 4c).

Rapid disease progression was observed in control animals, leading to their sacrifice by day 25 post-tumour inoculation. In contrast, 4Pm28ζ and 4Pm28ζN T cells exhibited potent anti-tumour activity, with no differences between groups, demonstrating no detrimental effect from the co-expression of the hNIS. Complete tumour regression was observed in all CAR T cell treatment groups, maintaining a tumour-free state for fifteen days before relapse (Fig. 4d, Fig. S6). Survival was significantly prolonged in all treatment groups compared to controls. Animals euthanised for tissue collection (n = 3/group) following the SPECT imaging session were censored in the survival analysis. No radiation toxicity was expected, as the estimated cumulative absorbed dose from repeated [⁹⁹ᵐTc]TcO₄⁻ and CT imaging was below 0.5 Gy [33]. No significant differences in survival were observed between CAR T treated groups (Fig. 4.e).

**Fig. 5:**
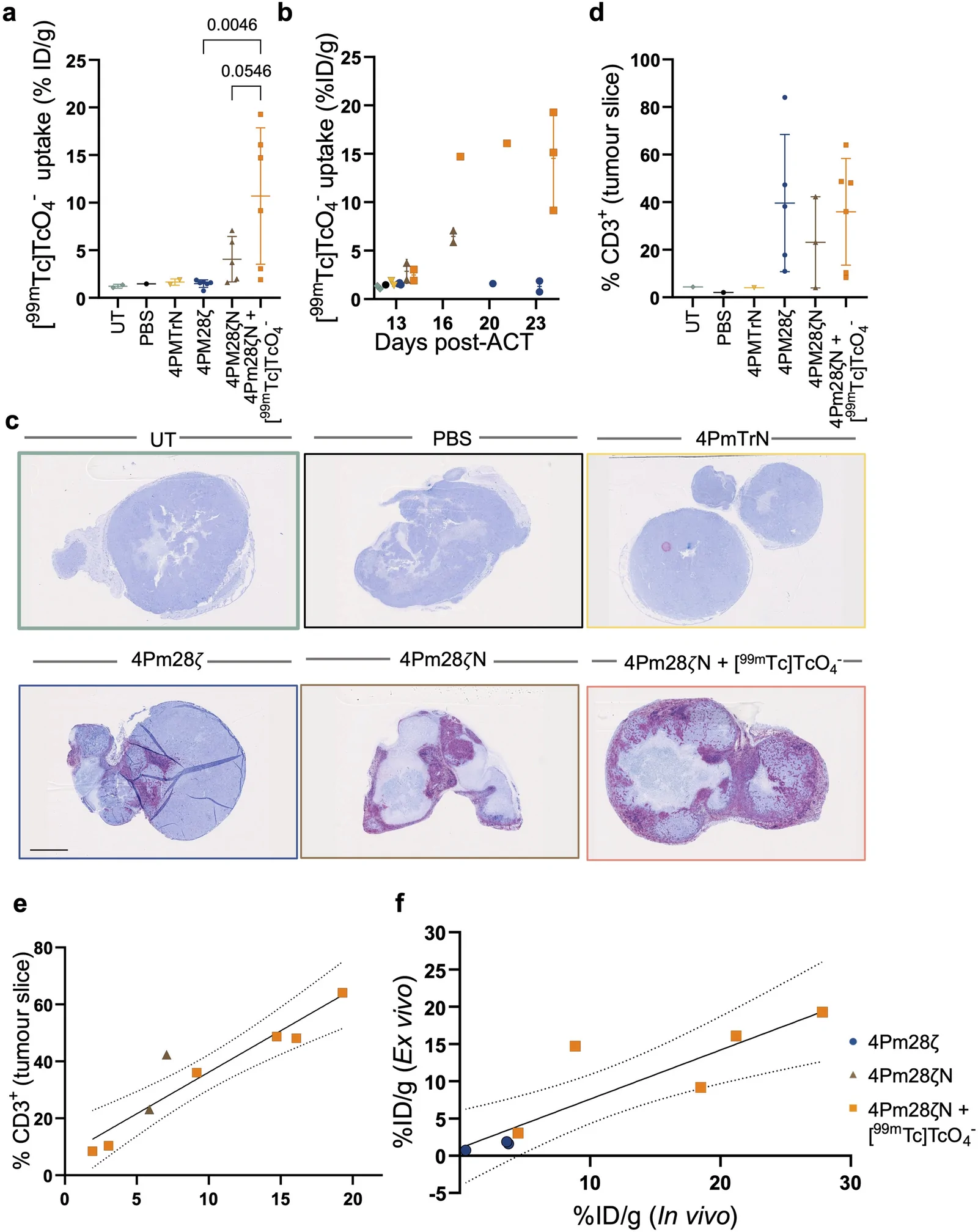
*Ex vivo* quantitation of CAR T cell infiltration. **A**) *Ex vivo* [^99m^Tc]TcO_4_^−^ uptake grouped by treatment. Ordinary one-way Anova. **B**) *Ex vivo* [^99m^Tc]TcO_4_^−^ uptake across treatment groups at the indicated time points post-ACT. **C**) Representative immunohistochemistry images of tumour sections stained for human CD3 from control (top row) and treated animals (bottom row). Scale bar: 2.5 mm. **D**) Quantification of CD3^+^ cells in tumour sections on day 13 post ACT. **E**) Correlation between *ex vivo* [^99m^Tc]TcO_4_^−^ tumour uptake and CD3^+^ cell density quantified using QuPath software. Pearson’s correlation r = 0.94. **F**) Correlation between *in vivo* SPECT-derived tumour uptae and *ex vivo* [^99m^Tc]TcO_4_^−^ tumour uptake quantified by gamma counting. Pearson’s correlation r = 0.88. For **E**) and **F**) solid line represents the linear regression best fit and dashed lines represent the 95% confidence bands. Graphs represent mean ± SD

*Ex vivo* analysis at various time points showed tumour-specific [^99m^Tc]TcO_4_^−^ uptake exclusively in 4Pm28ζN treated groups, with higher uptake observed in animals receiving repeated tracer administrations (Fig. 5a). Tumour uptake varied among animals but generally increased over time, reflecting progressive infiltration of CAR T cells post-ACT (Fig. 5b). Immunohistochemistry confirmed the absence of CD3^+^ T cells in control animals, while CAR T cell infiltration varied among treated animals, with comparable CD3^+^ T cells densities across all treated groups (Fig. 5c&d). A strong correlation (r = 0.94, p = 0.0005) was observed between tumour uptake quantified by gamma counting and CD3^+^ T cell infiltration quantified by immunohistochemistry in 4Pm28ζN treated animals (Fig. 5e), as well as between *in vivo* and *ex vivo* measurements of tumour uptake (r = 0.88, p = 0.0036) (Fig. 5f). These findings demonstrate that hNIS expression enables real-time tracking of CAR T cell infiltration without compromising anti-tumour activity.

### Dynamic SPECT/CT imaging of infiltrating CAR T cells *in vivo*

**Fig. 6:**
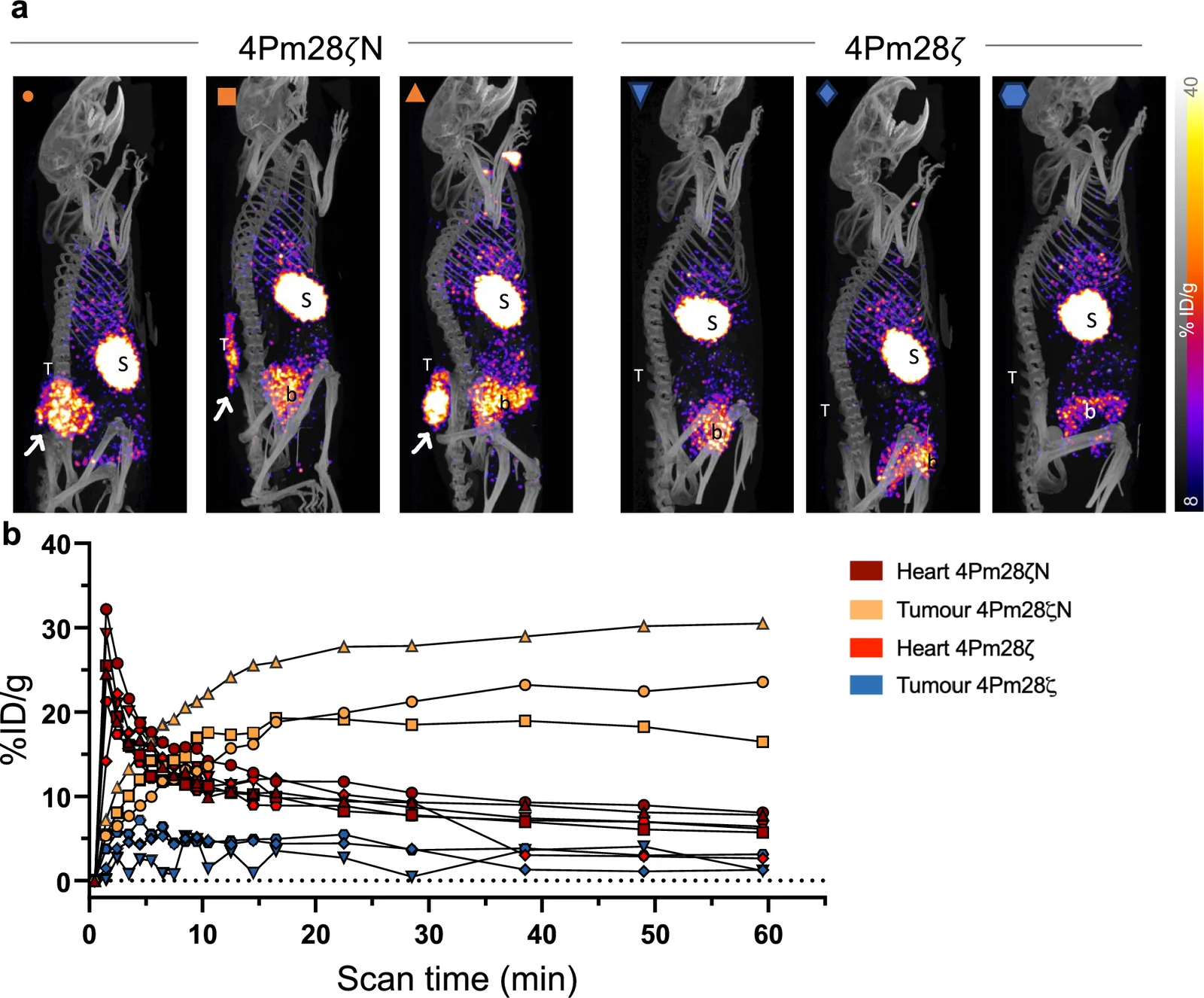
SPECT-CT imaging reveals infiltration of hNIS-expressing CAR T cells. **A**) Maximum intensity projection SPECT images depicting tumour-specific [^99m^Tc]TcO_4_^−^ uptake in 4Pm28ζN treated animals following administration of 10 MBq of [^99m^Tc]TcO_4_^−^, but not in non-hNIS expressing 4Pm28ζ CAR-T cells. White arrows point CAR T cell signal in the tumour. S = stomach, B = bladder, T = tumour. **B**) Time-activity curves of [^99m^Tc]TcO_4_.^−^ kinetics in heart and tumour of animals in **A**) acquire through dynamic SPECT/CT imaging. Uptake expressed as the percentage of injected radioactivity dose per gram of tissue (%ID/g)

Dynamic SPECT imaging revealed distinct tumour tracer kinetics between 4Pm28ζN and 4Pm28ζ treated animals. High tumour tracer uptake was observed exclusively in the 4Pm28ζN-treated cohort, consistent with functional hNIS expression, with no visible accumulation in the 4Pm28ζ group (Fig. 6a). The time-activity curves (TACs) derived from the heart regions demonstrated comparable tracer availability across both groups, showing a rapid peak in radioactivity shortly after tracer administration followed by a gradual decline over time, indicative of clearance from circulation (Fig. 6b). In 4Pm28ζ treated animals, tracer concentration in the tumours rapidly plateaued, suggesting passive perfusion with no evidence of active uptake by CAR T cells. In contrast, 4Pm28ζN-treated tumours exhibited a continuous rise in tracer uptake beyond perfusion levels with reduced efflux over time, consistent with reversible hNIS-mediated tracer accumulation in tumours containing viable hNIS-expressing CAR T cells. Tumour tracer uptake varied among individual 4Pm28ζN-treated animals, as reflected by the TACs (Fig. 6b).

**Fig. 7:**
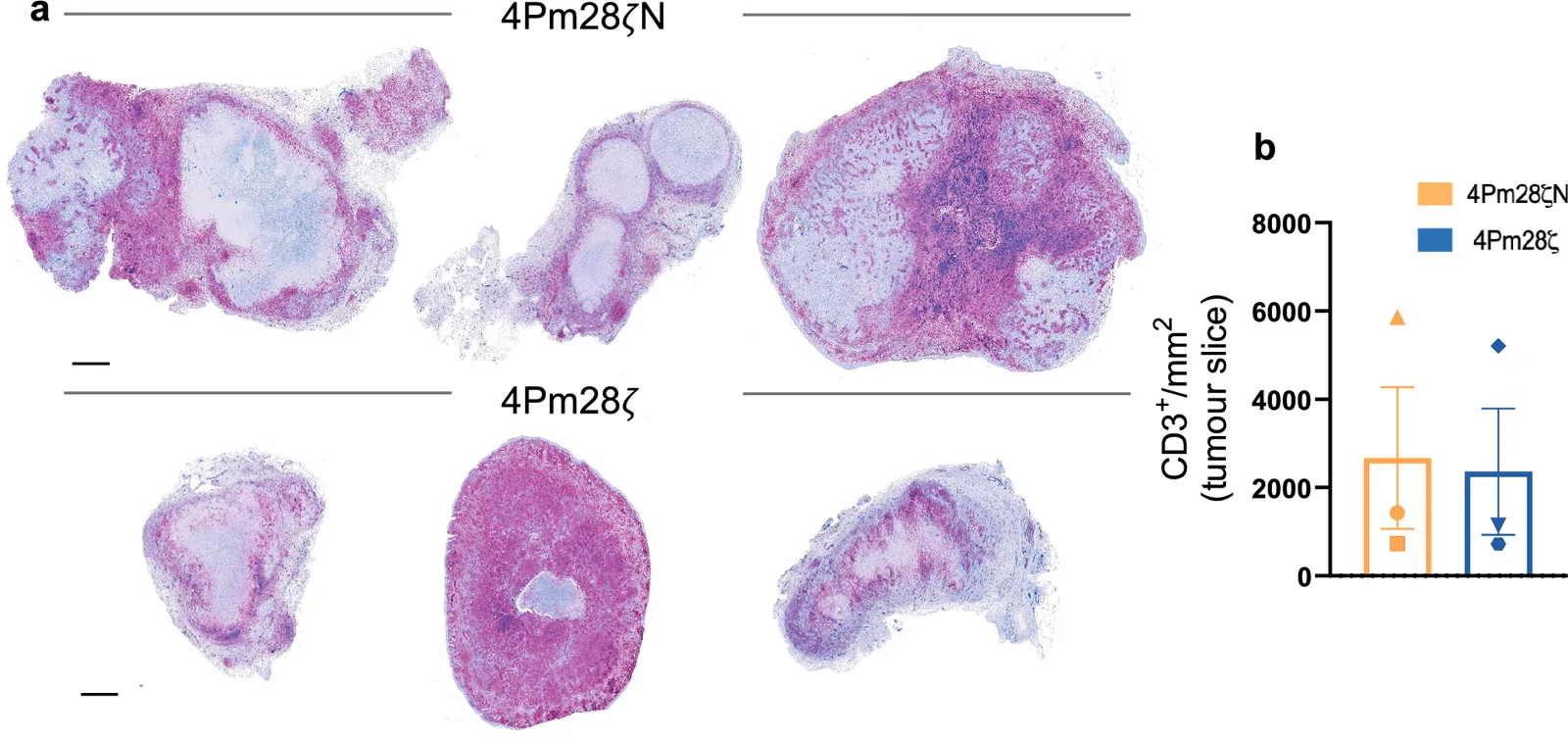
Immunohistochemistry demonstrates comparable intratumoral T cell infiltration. **A**) Paraffin embedded tumour slices were stained for human CD3 (pink) and CD31 (dark blue) in 4Pm28ζN (top) and 4Pm28ζ (bottom) treated animals. Scale bar: 1 mm. **B**) *Ex vivo* quantitation of CD3^+^ T cells from slices in **A**). n = 3/group. Graphs represent mean ± SD

**Fig. 8:**
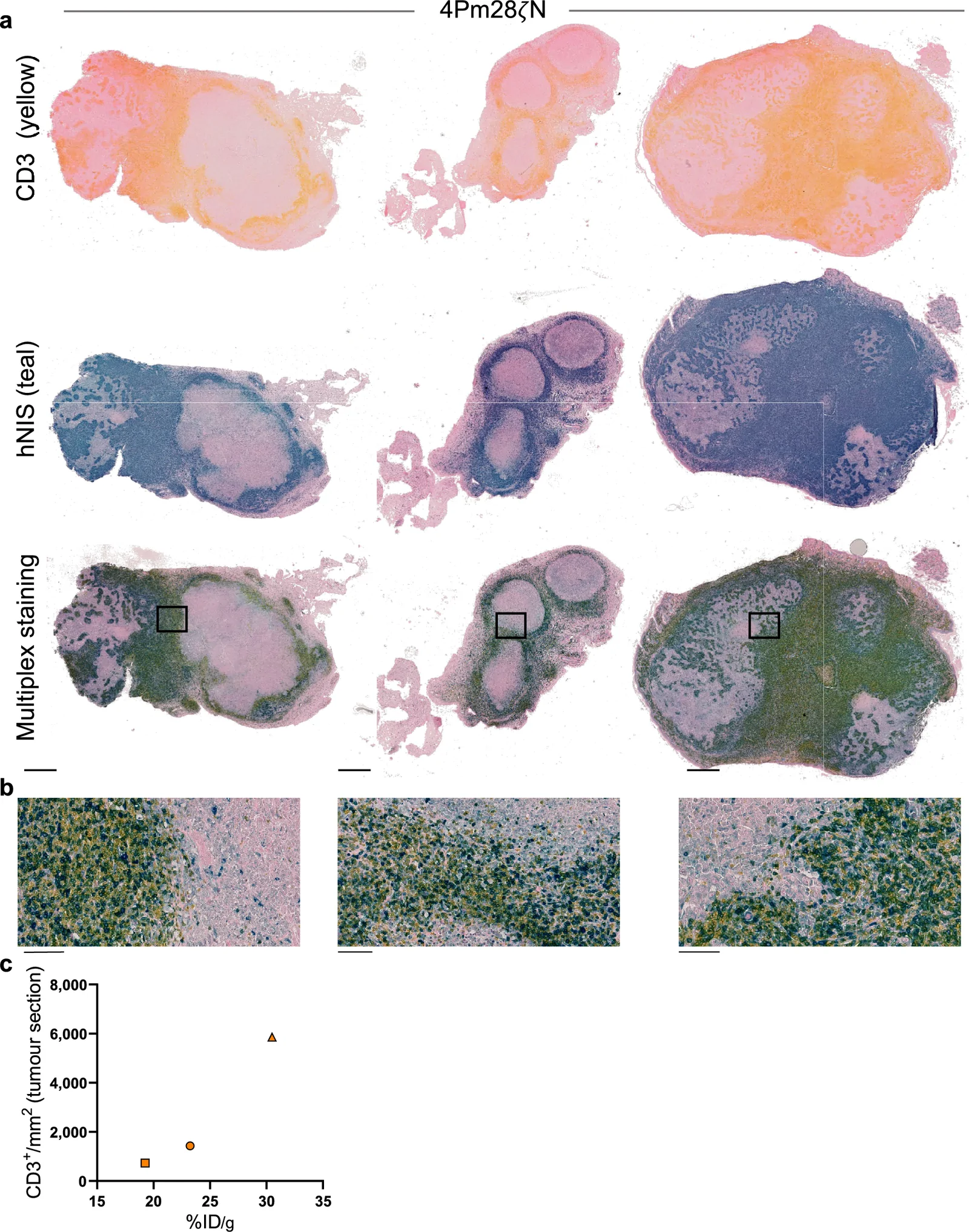
Multiplex immunohistochemistry confirms intratumoural CAR T cell identity. **A**) Paraffin embedded tumour slices were stained for human CD3 (yellow, top), human NIS (teal, middle), or simultaneous multiplex staining of CD3 and hNIS (bottom). Scale bar: 1 mm. **B**) High-magnification images showing the colocalisation of CD3 and hNIS. The location of magnification is shown by the black squares in **A**). Scale bar: 0.1 mm. **C**) Correlation between maximum tumour uptake quantified from time-activity curves in Fig. 6C and CD3^+^ T cell density from immunohistochemical analysis. n = 3/group

Despite the absence of detectable tracer accumulation in the 4Pm28ζ-treated group, *ex vivo* immunohistochemical analysis confirmed the presence of CAR T cells in both groups (Fig. 7a&b). Multiplex immunohistochemistry further demonstrated clear colocalisation of hNIS and CD3 within tumours from 4Pm28ζN-treated animals, confirming that hNIS expression is restricted to infiltrating CD3 ^+^ T cells and verifying their CAR T cell identity (Fig. 8a&b).

The inter-variability in tumour tracer uptake observed in 4Pm28ζN-treated animals (Fig. 6b), was also observed in CD3^+^ T cell density quantified by immunohistochemistry (Fig. 8c). These results are in line with the findings from *ex vivo* tracer biodistribution and T cell infiltration depicted in Fig. 5e, supporting the use of dynamic *in vivo* imaging as a reliable, non-invasive surrogate for assessing CAR T cell infiltration and NIS-mediated tracer retention.

**Fig. 9:**
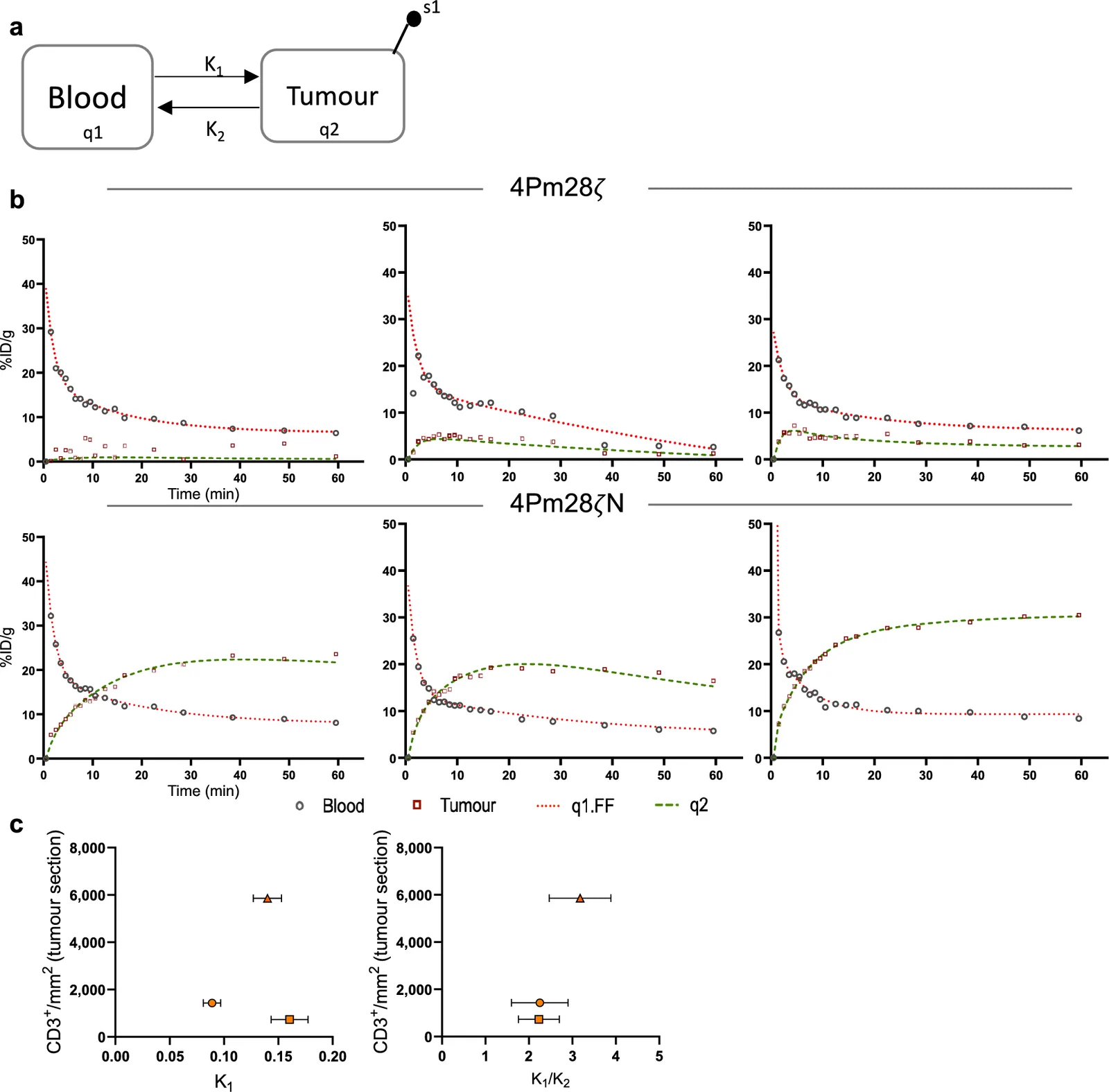
A two-compartmental model describes CAR T cell infiltration. **A**) Schematic of the two-compartment model. The blood compartment, q1, exchanges tracer with the tumour compartment, q2, at the rate defined by K_1_ and K_2_. **B**) Fit of the two-compartment model using SAAM II kinetic modelling software using the individual dynamic data from 4Pm28ζN and 4Pm28ζ treated animals. A forcing function (red dotted line) was applied to blood measurements (grey circles). The average tumour uptake is represented by the square dots, and the green dotted line represents the fitted model. **C**) Correlation between the *ex vivo* quantitation of the CD3^+^ cells in the tumour slices and K_1_ or K_1_/K_2_ ratio obtained from the two-compartment model. n = 3/group

To explore the mechanisms underlying the distinct tumour time-activity curves observed during the dynamic SPECT imaging, we applied compartmental kinetic modelling using SAAMII software. Both, two- and three-compartment reversible and irreversible models were evaluated in 4Pm28ζN-treated animals. Based on Akaike and Bayesian information criteria, a two-compartment reversible model provided the most appropriate description of the tumour TACs and was therefore used for all subsequent analyses (Fig. 9a&b).

Model-derived rate constants revealed differences in tracer kinetics between 4Pm28ζN and 4Pm28ζ-treated groups that were consistent with the observed TAC profiles. These differences reflect changes in tracer delivery and washout. On average, the tracer influx rate constant (K_1_) was 2 times higher in the 4Pm28ζN group (0.14 ± 0.014 min^−1^) compared to 4Pm28ζ tumours (0.07 ± 0.014 min^−1^), indicating superior initial uptake in the presence of hNIS-expressing CAR T cells. In parallel, the efflux rate constant (K_2_) was 3.3 times lowered in 4Pm28ζN tumours (0.066 ± 0.012 min^−1^) than in controls (0.218 ± 0.051 min^−1^), consistent with reduced efflux and delayed washout in tumours containing hNIS-expressing CAR T cells. Inter-animal variability in these parameters was observed and the rate constants for each individual animal are summarised in Table 1. SAAM II-derived fit -quality metrics and parameter uncertainty estimates are reported in Supplementary Table S1.

**Table 1:** Rate constant values estimated by two-compartment models fitted to individual animals

4Pm28ζN		4Pm28ζ	
K_1_ (min^−1^)	K_2_ (min^−1^)	K_1_ (min^−1^)	K_2_ (min^−1^)
0.089 (0.008)	0.0363(0.009)	0.0084 (0.003)	0.099 (0.005)
0.1604 (0.017)	0.0723 (0.013)	0.0774 (0.016)	0.257 (0.062)
0.1463 (0.0130)	0.0440 (0.009)	0.196 (0.005)	0.0448 (0.012)

^a^Standard deviation in brackets

Beyond visualising distribution, a potential advantage of kinetic modelling is the ability to distinguish NIS-mediated tracer uptake and washout kinetics from passive delivery while considering variables such as differences in tumour vascularity and injected doses of tracer. In this study, the influx rate K_1_ varied, likely reflecting differences in tumour perfusion, as observed in CAR control animals, plus differences in CAR-hNIS cell density. However, when accounting for washout using the K_1_/K_2_ ratio, equivalent to the distribution volume, the relationship with CAR T cell infiltration appeared more consistent (Fig. 9c). Although preliminary given the small cohort size, this observation suggests that metrics capturing the balance between tracer delivery and reversible retention (K_1_/K_2_ ratio) may provide a more robust measurement of CAR T cells presence than influx parameters alone, and warrants further investigation.

These data demonstrates that kinetic modelling adds interpretative value to dynamic SPECT imaging by clarifying the physiological origins of tumour-associated tracer uptake by distinguishing whether differences in tumour uptake arise from altered tracer delivery, reduced washout or a combination of both.

## Discussion

The limited efficacy of CAR T cell therapy in solid tumours remains a significant barrier to its widespread clinical application. Unlike haematological malignancies, solid tumours present additional challenges, including restricted T cell infiltration, immunosuppressive microenvironments, and heterogeneous antigen expression [9]. A major limitation in understanding and optimising CAR T cell therapies is the lack of robust, real-time methods to monitor cell distribution, persistence, and tumour engagement. Conventional methods such as peripheral blood monitoring and biopsy sampling provide only static, indirect measurements and do not capture the dynamic processes that govern CAR T cell efficacy [10, 14]. Our study addresses this challenge by demonstrating the feasibility of hNIS-mediated imaging for real-time, non-invasive tracking of CAR T cells *in vivo*, with kinetic modelling providing complementary quantitative insight into the source of tracer retention.

A key requirement for the clinical translation of any imaging reporter is its biological neutrality. *In vitro* characterisation confirmed that 4Pm28ζN CAR T cells-maintained proliferation, antigen-specific cytotoxicity, and cytokine secretion comparable to non-hNIS-expressing counterparts. Additionally, exposure to preclinically relevant doses of [^99m^Tc]TcO_4_^−^ did not induce DNA damage or impair function, supporting the safety of this approach for longitudinal tracking. These results are consistent with previous studies employing hNIS as a reporter gene for tracking adoptive cell therapies [12, 34], reinforcing its suitability for translational applications.

*In vivo* imaging further validated the utility of the NIS reporter gene for longitudinal monitoring of CAR T cell infiltration. SPECT/CT imaging demonstrated clear tumour-specific tracer accumulation in 4Pm28ζN-treated mice, whereas no uptake was observed in control 4Pm28ζ T cell-treated animals, despite confirmed CAR T cell presence by immunohistochemistry. These data demonstrate that tracer uptake is mediated by NIS, enabling selective visualisation. Both 4Pm28ζN and 4Pm28ζ T cells showed high *in vivo* potency, achieving complete tumour eradication by day 25 post-ACT, with animals staying tumour-free for 10–14 days before relapse. Repeated tracer administration did not compromise CAR T cell functionality or alter treatment outcomes, reinforcing the feasibility of this approach for longitudinal tracking.

This study leveraged dynamic SPECT imaging and tracer kinetic modelling to interpret the dynamic process contributing to tracer signal in the tumours. *In vitro* uptake assays demonstrated rapid radiotracer influx and efflux in response to changes in the extracellular tracer concentration supporting the reversibility of the system. *In vivo*, the application of a reversible two-compartment model allowed for the characterisation of tracer uptake and efflux. The markedly higher influx rate constant (K_1_) and lower efflux rate constant (K_2_) in 4Pm28ζN-treated tumours support that the increased signal was associated with hNIS-mediated radiotracer uptake and reduced tracer washout, rather than passive perfusion or vascular leakage.

Kinetic modelling offers a mathematical framework to account for physiological variables that static imaging cannot address, such as variability in tracer bioavailability due to administration differences or heterogeneity in tumour vascularity, and may offer insights into the pharmacokinetic and pharmacodynamic behaviour of CAR T cells *in vivo*. The influx rate constant (K_1_) acts as a proxy for tracer delivery to the tumour, reflecting local perfusion, which can vary significantly between subjects, and initial hNIS-mediated uptake by viable CAR T cells. Conversely, the efflux rate constant (K_2_) represents tracer washout from the tumour compartment reflecting reversible tracer exchange. The K_1_/K_2_ ratio, equivalent to the distribution volume, could therefore serve as a composite metric that balances tracer delivery against retention over time. In our cohort, this ratio appeared to offer a more stable surrogate metric for CAR T cell accumulation than uptake rates alone (Fig. 9c).

While this proof-of-concept study employed a single imaging timepoint in a small cohort, the identification of the K_1_/K_2_ ratio as a better parameter is a key step for translation protocols. Future applications of this model could incorporate longitudinal imaging to capture temporal changes in this ratio, providing dynamic, non-invasive measurements potentially associated with T cell expansion, persistence or clearance *in vivo*.

We observed a lack of correlation between surface NIS expression and radiotracer uptake across different donors, suggesting that surface abundance alone does not fully predict functional NIS activity. This observation is consistent with previous reports demonstrating that NIS functionality is modulated by factors beyond copy number, including correct intracellular trafficking, post-translational glycosylation, membrane potential, and sodium gradient regulation [22]. Glycosylation plays a critical role in stabilising NIS conformation and enhancing transport efficiency. Hypoglycosylated NIS can reach the plasma membrane but exhibits reduced iodide transport capacity [14, 35]. While we confirmed NIS surface localisation by flow cytometry, glycosylation status was not assessed in this study, which may contribute to the observed donor-to-donor variability. Other authors have reported that above a certain threshold, further increases in NIS expression do not proportionally enhance uptake [36], reflecting the influence of other factors beyond copy number, which may relate to differences in ion channel expression, cell metabolic fitness, and iodide efflux regulation. Despite the observed donor-to-donor differences in NIS surface expression and uptake levels, our study shows that modest levels of *in vitro* uptake (~ 7% in 4Pm28ζN cells, equivalent to 0.233 Bq/cell) were sufficient to detect strong tumour-specific tracer signal *in vivo*. These observations highlight the importance of performing functional assays before CAR T cell infusion.

Despite these promising results, several limitations should be acknowledged. First, the heart ROI was used as a surrogate for blood input rather than a dedicated left ventricular blood-pool ROI, which may introduce variability in kinetic modelling parameters. No arterial sampling or partial-volume correction was applied. While this approach enabled fully non-invasive dynamic imaging, it may introduce spillover and partial-volume effects, which are recognised limitations of image-derived input function methodologies. Although the fitted parameters showed finite confidence intervals, formal model stability by repeated fitting from different starting values, bootstrap resampling or sensitivity analysis were not performed in this exploratory study. More refined methods, such as image-derived input functions from specific vascular structures, and larger longitudinal cohorts could improve accuracy. Second, while flow cytometry could, in principle, provide precise quantification of CAR T cell numbers, our attempts using dissociated tumour tissues yielded poor and unreliable detection of CAR and hNIS expression. In contrast, immunohistochemistry provided robust staining and enable spatial confirmation of CAR T cell infiltration, but quantitation is influenced by spatial heterogeneity. Nevertheless, the strong correlation observed between CD3^+^ T cell density quantified by immunohistochemistry and *ex vivo* tracer uptake measured by gamma counting indicates that CD3^+^ quantitation from a central section provides a reasonable surrogate measurement for overall T-cell burden in this model. Multiplex immunohistochemistry demonstrated colocalisation of hNIS and CD3 confirming that infiltrating T cells corresponded to transduced CAR T cells, offering spatial information that more closely aligns with the SPECT imaging data. Lastly, while the study was conducted in a small cohort as a proof-of-concept with limited statistical power, the magnitude of the kinetic differences between treated and control tumour suggests that the biological signal from hNIS-mediated retention is robust enough to be detected even in limited sample sizes. Future studies combining larger cohorts, longitudinal imaging and addition endpoint markers of CAR T-cell activation, proliferation and exhaustion will be required to validate the reproducibility and biological significance of the proposed modelling framework and reflect the functional CAR T-cell state in addition to localisation.

A major advantage of this approach is its broader applicability across both preclinical and clinical imaging studies. Although dynamic PET remains the most established modality for quantitative kinetic imaging in many clinical settings, the objective of the present study was to establish a preclinical proof-of-concept using [^99m^Tc]TcO_4_^−^, a widely available and accessible hNIS-compatible SPECT tracer. In this context, the GP-RM SPECT collimator provided sufficient temporal and quantitative recovery for small-animal modelling, as supported by phantom-based accuracy quantitation (Fig. S7). Importantly, the underlying kinetic framework is modality-agnostic and can be adapted for other radiotracers and imaging modalities, such as PET imaging with [^18^F]Tetrafluoroborate, which offers improved sensitivity and spatial resolution [37–39]. Given the growing interest in PET-based cell tracking for clinical applications and the advent of Total Body PET scanners [40], which are faster and more sensitive, the potential for using kinetic modelling for CAR T cell monitoring and quantitation has never been higher. Other authors have previously discussed the possibility of quantifying NIS mediated uptake of radiopharmaceuticals at a single point in time post injection [41], but this approach is limited by the reversible pharmacokinetics of [^99m^Tc]TcO_4_^−^ or [^18^F]Tetrafluoroborate, which make definition of an appropriate time point challenging. Kinetic modelling, by accounting for these time-dependent changes, can mitigate this limitation and provide a more robust framework for both preclinical studies and clinical translation.

In conclusion, this study presents the first application of NIS-based imaging combined with kinetic modelling to investigate CAR T cell infiltration *in vivo*. Dynamic SPECT imaging enabled non-invasive assessment of T cell dynamics while preserving therapeutic efficacy, while kinetic modelling provided mechanistic insight into the process leading to tracer signal, distinguishing between tracer delivery and transporter-mediated retention. Its adaptability to clinically relevant tracers and imaging platforms has the potential to enhance the monitoring and understanding of CAR T cell therapies in solid tumours.

## Conclusions

This study demonstrates the feasibility of using a reversible two-compartment kinetic model to interpret hNIS-based SPECT imaging of infiltrating CAR T cells. Our findings show that dynamic modelling distinguishes active hNIS-mediated uptake, characterised by increased tracer influx (K_1_) and reduced efflux (K_2_), and accounts for variable tracer bioavailability, addressing a key limitation of static imaging approaches. While preliminary, the K_1_/K_2_ ratio showed potential as a more consistent image-derived parameter associated with CAR T cell presence than uptake rates alone. This approach establishes a preliminary quantitative framework for dynamic CAR T cell imaging providing additional kinetic information beyond static uptake measurements, and supports further investigation of kinetic modelling approaches for longitudinal cell tracking in solid tumours.

## Materials and methods

### Plasmid DNA

CAR constructs were expressed using the SFG retroviral vector. The previously generated second-generation 4P28ζN and 4PTrN CAR constructs [21] were modified to clone a fused myc epitope tag to the CD28 hinge domain using NEBuilder HiFi DNA Assembly Kit as per manufacturer instructions (NEB, Massachusetts, USA). These resulting new constructs, 4Pm28ζN and 4PmTrN, were generated alongside a bicistronic control CAR, 4Pm28ζ, lacking hNIS. Assembly reactions were transformed into NEB 5-alpha competent E.Coli as per the manufacturer’s instructions. DNA was extracted from ampicillin resistant bacterial colonies using the E.Z.N.A Plasmid DNA Mini Kit II (Omega Bio-Tek, Vermont, USA).

### Cell lines

PC3-LN3 (PL) (Professor Sue Eccles, Institute of Cancer Research), PC3-LN3-PSMA (PLP), PL-Luciferase dTomato (PL-LT), PLP-LT (Fig. S2) and HEK293T were cultured in Dulbecco’s Modified Eagle Medium (DMEM) (Gibco, Thermo Fisher Scientific, Massachusetts, USA) supplemented with 10% Foetal Bovine Serum (FBS) (Sigma-Aldrich, UK) and 1% L-Glutamine (Sigma-Aldrich, UK). HEK 293 T cells were obtained from the European Collection of Authenticated Cell Cultures. All cell lines were cultured at 37 °C in 5% CO_2_ and monitored for mycoplasma contamination.

### Human samples and PBMC isolation

Peripheral blood mononuclear cells (PBMCs) were isolated from healthy volunteers, aged 18–64 years, with National Health Service Research Ethics Committee approval (reference 09/H0804/92 and 18/WS/0047). Blood was layered over Ficoll-Paque (GE Healthcare, UK) and centrifuged at 800 g for 35 min. The PBMC fraction was washed, resuspended in Roswell Park Memorial Institute (RPMI) 1640 (Gibco, Thermo Fisher Scientific, Massachusetts, USA) supplemented with 5% Human serum (Sigma-Aldrich, USA) and 1% L-Glutamine, and activated with 5 μg/ml Phytohaemagglutinin-L (PHA-L) (Sigma-Aldrich, UK) for 48 h. Recombinant human IL-2 (100 IU/ml, R&D systems, Minneapolis, USA) was added after 24 h.

### Retroviral production and CAR T cell transduction

Retroviral supernatants were generated by transfecting HEK293T cells with the CAR plasmid, pEQ-Pam3, and RD144 using GeneJuice (Sigma-Aldrich, UK). Viral supernatants were collected at 48 and 72 h, snap-frozen, and stored at − 80 °C. PBMCs were transduced on RetroNectin-coated (11 μg/ml) (Takara, California, USA) non-tissue culture-treated 6-well plates. Viral supernatant was preloaded onto plates, followed by the addition of PBMCs (1 × 10⁶/well) and 3 ml of viral supernatant plus 100 IU/ml of IL-2. Transduced cells were maintained in RPMI supplemented with 5% Human serum and recombinant human IL-4 (3 ng/ml, Peprotech, UK), while untransduced cells received IL-2 (100 IU/ml). Cells were maintained at a concentration of 2 × 10^6^ cells/ml.

### Cytotoxicity assay

For endpoint cytotoxicity assays, 1 × 10^4^ target cells (PL, PLP, PL-LT, or PLP-LT) were seeded in 96-well plates and co-cultured with CAR T cells at specified effector-to-target (E:T) ratios for the indicated duration. Tumour viability was assessed using either 500 μg/ml of 3-(4,5-dimethylthiazol-2-yl)−2,5-diphenyltetrazolium bromide (MTT) (Sigma-Aldrich) or real-time imaging with an Incucyte® S3 Live-Cell Analysis System (Sartorius, Germany). MTT crystals were dissolved in DMSO and absorbance was measured at 590 nm using a FLUOstar Omega plate reader (BMG LabTech, Germany). For real-time imaging four images per well were acquired every 2 h for 70 h using a 10 × magnification. Incucyte® fluorescence signal changes were normalised to baseline and analysed over time.

### Cytokine release assay

Supernatants from CAR T-cell and target cell co-cultures were collect after 24 for IL-2 ((Invitrogen, Thermo Fisher Scientific, UK) and 48 h for IFNγ (R&D systems, UK) quantification by enzyme-linked immunosorbent assay (ELISA) as per the manufacturer’s instructions.

### Flow cytometry analysis

For surface marker staining, 1 × 10^5^ viable cells were incubated with Zombie Near Infrared (NIR) Live/Dead stain (1:1000, BioLegend, UK) for 15 min at RT in the dark, followed by staining with PE-conjugated anti-MYC (9B11, 1:20, Cell Signalling, UK) or anti-hSCL5A5-AF647 (1:10, R&D Systems, UK) for 30 min at 4°C. Cells were washed and resuspended in staining buffer (PBS + 2% FBS) ready for acquisition. Single stained compensating controls were prepared with 1 drop of UltraComp eBeads (Invitrogen) and 1 μl of the relevant antibody. Quantum R-PE and AF647 MESF beads (Bang Laboratories, USA) were used to quantify surface CAR and hNIS expression, respectively. Cell viability was assessed by staining for Annexin V-PE (1:20, BioLegend, UK) and Zombie NIR staining in Annexin V binding buffer. DNA damage was quantified following exposure to [^99m^Tc]TcO_4_^−^. Cells were fixed in 90% ethanol, stored at − 20 °C, and stained with phosphorylated γH2AX-PE staining (1:100, BioLegend, UK) or Mouse IgG_1_k-PE isotype (BioLegend, UK) for 30 min at RT in the dark.

Samples were acquired using a BD LSRFortessa Flow Cytometer and analysed using FlowJo software (BD, New Jersey, USA).

### In vitro [^99m^Tc]TcO_4_^−^ assays

[^99m^Tc]TcO_4_^−^ was provided by the Barts Health Radiopharmacy, Barts Health NHS Trust, London, from a generator, which had been eluted in the last 24 h.

[^99m^Tc]TcO_4_^−^ assay: 3 × 10^5^ viable cells were incubated in triplicate with 0.1 MBq [^99m^Tc]TcO_4_^−^ (0.33 MBq/ml) in PBS ± 10 mM sodium perchlorate (Sigma-Aldrich, UK) for 30 min at 37 °C, 5% CO₂. Cells were washed in 1 ml of ice-cold PBS, pelleted, and radioactivity quantified using an automatic gamma counter (LKB Wallac 192 Compugamma CS). Uptake was expressed as intracellular uptake percentage of total radioactivity added:

$$\begin{aligned}&\mathrm{U}\mathrm{p}\mathrm{t}\mathrm{a}\mathrm{k}\mathrm{e} (\mathrm{\%})\hspace{0.17em}=\hspace{0.17em}[\mathrm{C}\mathrm{o}\mathrm{u}\mathrm{n}\mathrm{t}\mathrm{s} \mathrm{p}\mathrm{e}\mathrm{r} \mathrm{m}\mathrm{i}\mathrm{n} (\mathrm{c}\mathrm{e}\mathrm{l}\mathrm{l} \mathrm{p}\mathrm{e}\mathrm{l}\mathrm{l}\mathrm{e}\mathrm{t}\mathrm{s})\\&/ \mathrm{C}\mathrm{o}\mathrm{u}\mathrm{n}\mathrm{t}\mathrm{s} \mathrm{p}\mathrm{e}\mathrm{r} \mathrm{m}\mathrm{i}\mathrm{n} (\mathrm{s}\mathrm{t}\mathrm{a}\mathrm{n}\mathrm{d}\mathrm{a}\mathrm{r}\mathrm{d}\mathrm{s})]\hspace{0.17em}\times \hspace{0.17em}100\end{aligned}$$

Time-Dependent Uptake and Efflux: For uptake kinetics, cells were incubated with [^99m^Tc]TcO_4_^−^ for 10–180 min at 37 °C, 5% CO_2_, washed at each time point and incubated on ice until the last time point. Maximum uptake was normalised to 100%. For efflux assays, cells were exposed to 0.1 MBq [^99m^Tc]TcO_4_^−^ (0.33 MBq/ml) and incubated for 30 min at 37 °C, 5% CO_2_. Following incubation, cells were resuspended in medium containing 88 kBq/ml of [^99m^Tc]TcO_4_^−^ (mimicking *in vivo* blood concentration conditions) and radioactivity measured at intervals up to 180 min. Uptake at 0 min was normalised to 100%.

Radiation-Induced DNA Damage: 5 × 10^5^ cells were exposed to [^99m^Tc]TcO_4_^−^ (0–25 MBq/ml) for 30 min at 37 °C, 5% CO₂. Cells were then incubated in fresh media until fixation with ice-cold ethanol and stored at − 20 °C for γH2AX staining.

### In vivo studies

All *in vivo* experiments were approved by the Queen Mary University London Animal Welfare Ethical Review Body (AWERB) and licenced under the UK Home Office Animals (Scientific Procedures) Act 1986 (ASPA) as specified in project licence number PP6127261 and reported in accordance with the ARRIVE guidelines.

*M*ale NOD scid gamma (NSG JAX strain, NOD.Cg-*Prkdc*^*scid*^* Il2rg*^*tm1Wjl*^/SzJ), aged 6–8 weeks, were purchased from Charles River (Harlow, UK). Mice were housed up to 6 animals per cage in individually ventilated cages under specific pathogen-free conditions. Animals were maintained on a 12-h light/dark cycle, with unrestricted access to water and standard rodent chow. Environment enrichment (wood shavings and a cardboard tubes) was provided in all cages. Animals were allowed to acclimatise for one week prior to any experimental procedures. A systematic block design was used for cage rack placement to minimise confounders associated with the microenvironment. A total of 48 animals were used. NSG mice were selected because their severe immunodeficiency allows the engraftment of human tumour cells and adoptive transfer of human CAR T-cells without immune rejection.

PSMA xenograft model: 2.5 × 10^5^ PLP-LT cells were inoculated subcutaneously in the flank area. After 12 days, mice were randomly assigned to experimental groups using a computer-generated randomisation list, with comparable tumour size across groups. On day 12, 2 × 10^6^ CAR T cells (4Pm28ζN, 4Pm28ζ; n = 10) or control treatments (PBS, UT, 4PmTrN; n = 5) were administered intravenously in 200 μl of PBS by blinded operators. As an exploratory proof-of-concept study, a statistical power calculation was not performed. No predefined exclusion or inclusion criteria was applied, and no animals were excluded from the analysis.

SPECT-CT imaging: Images were acquired using a multi-modal SPECT/CT scanner (VECTor^6^ MiLabs) fitted with a 1.5 mm multi-pinhole (GP-RM) collimator. Quantification accuracy for the GP-RM collimator used in this study was performed using a point source phantom containing 20 MBq [^99m^Tc]TcO_4_^−^ placed inside a water jacket and imaged repetitively across a range of expected activities and dynamic frame durations. Recovered activity was compared with expected activity and expressed as percentage deviation, calculated as [(expected-measured)/expected] × 100 with an acceptance criterion of 5% deviation. These data were used to define the quantitative range of the dynamic SPECT imaging protocol used in this study (Fig. S7). Mice allocated to the imaging cohort were kept under 2% isoflurane in air throughout the imaging session and received heat support from an integrated heating source in the scanner bed. Respiration and bed temperature were monitored and adjusted using BioVet sensors (m2m Imaging; Newark, USA). A counterbalanced reversed-order sequence was followed at each imaging time point to minimise confounders associated with the time of day and order of measurements. Dynamic SPECT imaging was acquired in list mode (30 s/frames, 78 frames) with 2-bed positions to contain the heart and the tumour xenograft within the field of view. 10 MBq of [^99m^Tc]TcO_4_^−^ was injected intravenously on the scanner bed after an initial background frame. For static SPECT imaging, animals were injected 10 MBq of [^99m^Tc]TcO_4_^−^ 30 min prior to imaging. SPECT imaging was acquired in list mode (30 min/frame, 1 frame) with 2-bed positions to contain the whole body. A whole-body CT scan was acquired for anatomical co-registration following SPECT imaging. SPECT/CT data were reconstructed using the integrated MiLabs Rec 11.00 software. The CT images were reconstructed at 80 μm^3^. SPECT frames were binned (10 × 1 min, 3 × 2 min, 2 × 6 min, 2 × 10 min, 2 × 15 min for dynamic imaging or 1 × 30 min for static scan) and reconstructed using the SROSEM algorithm (0.8 mm^3^, 128 subsets, 9 iterations), registered to the CT and corrected for attenuation, and radioactivity decay. Images were analysed using VivoQuant software (Perceptive Discovery, USA), with ROI’s defined for the heart and tumour using CT anatomical data. Radiotracer uptake was normalised to the injected dose and expressed as percentage of the injected radioactivity dose (%ID) or percentage of the injected radioactivity dose per gram of tissue (%ID/g).

#### Welfare

Mice were monitored daily for general health. Weight was monitored every 7 days. Tumour growth was monitored by calliper measurements by blinded operators every 2–3 days. Tumour volume was calculated using digital callipers as length × width^2^ × (π/6). Humane endpoints included weight loss exceeding 15%, tumour ulceration over 4 mm, signs of distress, or tumour size exceeding 1.44 cm2. No unexpected adverse effects were observed during the study.

#### Compartmental modelling

A two-compartment reversible model was assumed to describe the [^99m^Tc]TcO_4_^−^ uptake and clearance from the bloodstream into a tumour and its subsequent efflux back into circulation.

Full compartmental analysis was performed in SAAM II v2.3.1.1. (The Epsilon Group, Washington, USA) to solve first order differential equations and estimate the kinetic parameters, K_1_ and K_2_. The tracer concentration in the bloodstream was quantified from the heart ROI, while tumour tracer concentration was obtained from the tumour ROI.

To account for the missing excretion and distribution of the tracer to other organs from the central blood compartment, a forcing function (FF) was applied, fitting a two-phase exponential decay equation to the blood dynamic data, before estimation of the tumour kinetic parameters.

Model fit quality was assessed using SAAM II statistics such as, objective function, scaled data variance, Akaike information criterion and Bayesian information criterions, alongside visual inspection of the measured and fitted time-activity curves. Parameter uncertainty was assessed by the software’s reported standard deviation, coefficient of variation, and 95% confidence intervals for K_1_ and K_2_.

The resulting data were plotted using Prism v.9 (GraphPad).

#### Ex vivo biodistribution

At the indicated time points, animals were sacrificed and organs of interest were immediately dissected and transferred to pre-weighed scintillation tubes. Radioactivity was quantified using a gamma counter, calibrated with known standards, as described above.

#### Immunohistochemistry

Tumours were fixed in 10% neutral buffered formalin (Sigma-Aldrich, UK) for 24 h, paraffin-embedded, and sectioned at 4 μm. For each tumour, one representative central section was processed by blinded operators. Immunohistochemistry staining was performed by the BCI/CRUK Pathology Facility, Barts Cancer Institute using an automated Ventana Discovery XT machine (Roche Diagnostics Ltd., Indiana, USA). Following deparaffinization and standard heat-induced epitope retrieval in Tris–EDTA buffer (pH 7.8), sections were incubated with anti-CD31 (Abcam,UK ab28364, 1:200), anti-CD3 (Abcam, UK, ab11089, 1:150), anti-hNIS (Proteintech, Illinois, USA, 24,324–1-AP, 1:200) or both for multiplex staining. Detection involved a secondary rabbit anti-rat Ab antibody (Vector Laboratories Inc., Californica, USA, AI-4001, 1:300) or OmniMap anti-Rabbit HRP (Roche Diagnostics Ltd. USA, 760–4311), respectively. For multiplex staining, DISCOVERY Teal HRP Kit (Roche Diagnostics, USA, 670–247) and DISCOVERY Yellow HRP kit (Roche Diagnostics, USA, 760–250) were used. Red counterstain II (Roche Diagnostics, USA, 780–2218) was used as a counterstain. Slides were scanned at 40 × magnification using a Nanozoomer s210 slide scanner (Hamamatsu, Japan). Quantification of CD3⁺ cells was performed using QuPath software. Tissue sections were threshold-optimised to differentiate positive from negative staining, and batch analysis was performed using the ‘positive cell detection’ tool to quantify CD3⁺ cells across the entire tissue section.

### Statistics

Statistical significance was determined as stated in the Figure legends. Statistical significance was determined at p < 0.05. Data are presented as mean ± SD. Statistical analysis was performed with Prism 9 software (GraphPad Software, California, USA).

## Supplementary Information

Additional file: 1 Proposed two-compartment model built in SAAM II. Model schematic showing tracer exchange between blood and tumour compartments at rates defined by K_1_ and K_2_. The blood compartment was fixed with a forcing function and the model was fitted to the quantified tumour signal, s1. The flow of tracer between the central and tissue compartments is defined by the differential equation. Related to methods

Additional file: 2 Characterisation of target tumour cells. Flow cytometry contour plots of target cells depicting PSMA and TdTomato cell surface expression. PL = PC3-LN3. PLP = PC3-LN3-PSMA. LT = Luciferase TdTomato. Related to Figure 3

Additional file: 3 Antigen-specific cytotoxicity of CAR T cells when co-cultured with target cells. Real time tumour cell viability after co-culture of CAR T cells or untransduced T cells with tumour cells at different effector-to-target ratios. Data acquired using Incucyte®. n=4 donors/group. Graphs represent mean cytotoxicity from 4 donors

Additional file: 4 *In vivo* [^99m^Tc]TcO_4_^-^ uptake kinetics and *ex vivo *biodistribution. A) Time-activity curve from dynamic SPECT/CT imaging of the heart. Three animals were imaged at day 12 and day 22 post tumour inoculation. n=6 samples/data point. B) *Ex vivo* biodistribution at day 22 post-tumour inoculation, inset shows zoom-in on tumours. n=3 animals. Related to Figure 3

Additional file: 5 High transduction efficiency of CAR T cells. A) Representative flow cytometry histograms showing transduction efficiency, assessed by surface CAR and by reporter gene expression on day 13 of expansion. Two million CAR^+^ T cells were injected in NSG mice the following day, as illustrated in the schematic in Figure 4A. Related to Figure 4

Additional file: 6 CAR T cell treatment shows enhanced tumour control. Calliper measurements of individual tumour volumes. The dashed line represents the day of adoptive cell transfer. n=10, n=5. Graphs represent mean ± SD. Related to Figure 4

Additional file: 7 Phantom-based quantification accuracy for the GP-RM collimator. A point source phantom containing known activity levels was placed in a water jacket and repetitively imaged at the centre FOV at 5- and 30-seconds, 1 or 5 minutes. Percentage of deviation was calculated as [(expected activity-measured activity/expected activity] x 100. Dashed lines indicated the predefined acceptable deviation limit established at 5 % 

Supplementary Material 8

## Data Availability

All data generated or analysed during this study are included in this published article and its supplementary information files.

## Abbreviations

ACT: Adoptive cell transfer
CAR: Chimeric antigen receptor
CT: Computerised tomography
IL-2: Interleukin-2
IL-4: Interleukin-4
IFNγ: Interferon-gamma
MFI: Median fluorescence intensity
NaClO_4_: Sodium perchlorate
NIS: Sodium iodide symporter
PET: Positron emission tomography
PL: PC3-LN3
PLP: PSMA expressing PC3-LN3
PSMA: Prostate-specific membrane antigen
SD: Standard deviation
SPECT: Single-photon emission computed tomography
[^99^^m^Tc]TcO_4_^−^: Technetium-99 m pertechnetate
TAC: Time-activity curve
TME: Tumour microenvironment
UT: Untransduced
