# Supplementary material for: Dynamic SPECT imaging kinetic modelling characterises tracer influx and efflux in tumour-infiltrating CAR T cells
Source: EJNMMI Res. 2026 Aug 29;16:160. doi: 10.1186/s13550-026-01492-y (PMC13768539; doi:10.1186/s13550-026-01492-y)
Supplement: Supplementary file 1 [file 13550_2026_1492_MOESM1_ESM.pdf]

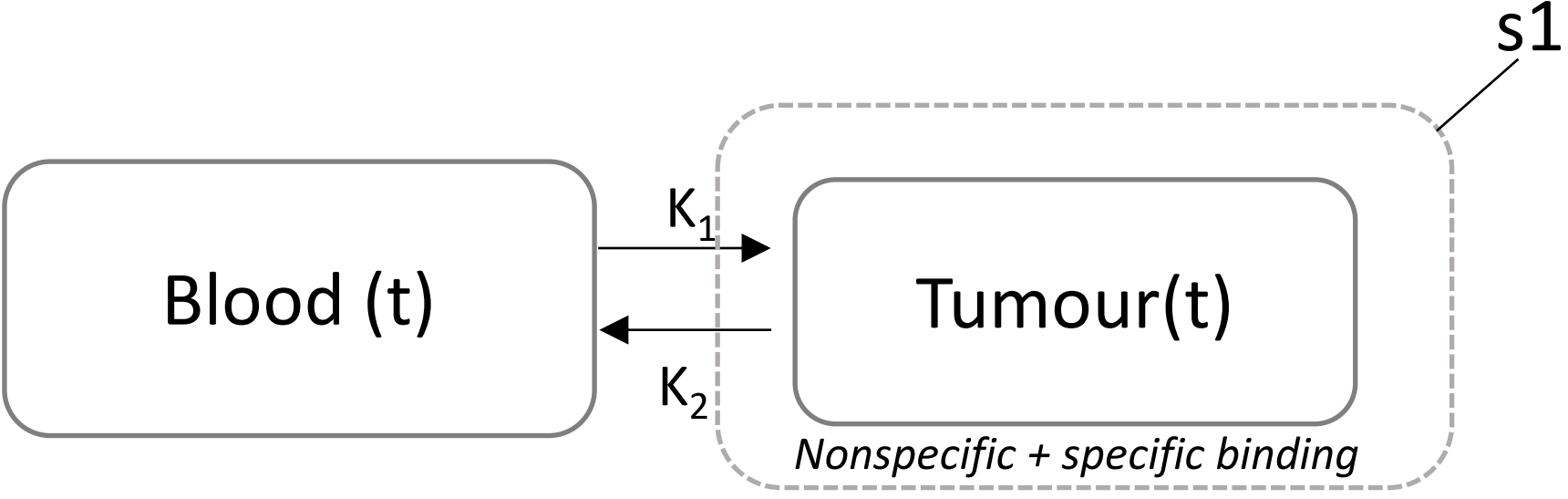

$$\frac{dC_{tumour}(t)}{dt} = \mathrm{K}_1 C_{\mathrm{blood}}\,(t) - \mathrm{k}_2 C_{\mathrm{tumour}}\,(t)$$
