# Supplementary material for: Dynamic SPECT imaging kinetic modelling characterises tracer influx and efflux in tumour-infiltrating CAR T cells
Source: EJNMMI Res. 2026 Aug 29;16:160. doi: 10.1186/s13550-026-01492-y (PMC13768539; doi:10.1186/s13550-026-01492-y)
Supplement: Supplementary file 2 [file 13550_2026_1492_MOESM2_ESM.pdf]

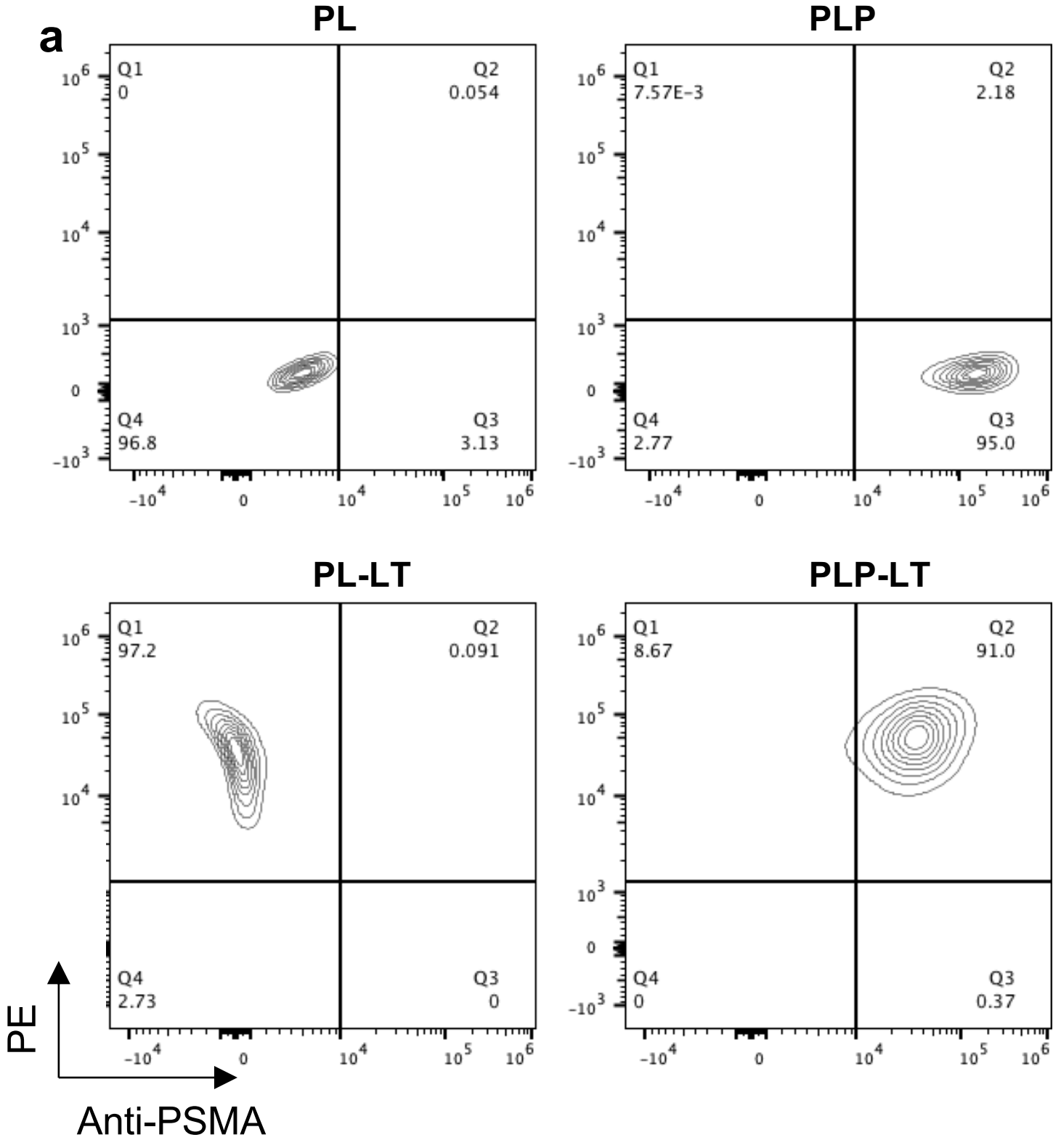

a
PL
PLP
PL-LT
PLP-LT
Q1
0
Q2
0.054
Q4
96.8
Q3
3.13
Q1
7.57E-3
Q2
2.18
Q4
2.77
Q3
95.0
Q1
97.2
Q2
0.091
Q4
2.73
Q3
0
Q1
8.67
Q2
91.0
Q4
0
Q3
0.37
PE
Anti-PSMA
