# Supplementary material for: Dynamic SPECT imaging kinetic modelling characterises tracer influx and efflux in tumour-infiltrating CAR T cells
Source: EJNMMI Res. 2026 Aug 29;16:160. doi: 10.1186/s13550-026-01492-y (PMC13768539; doi:10.1186/s13550-026-01492-y)
Supplement: Supplementary file 3 [file 13550_2026_1492_MOESM3_ESM.pdf]

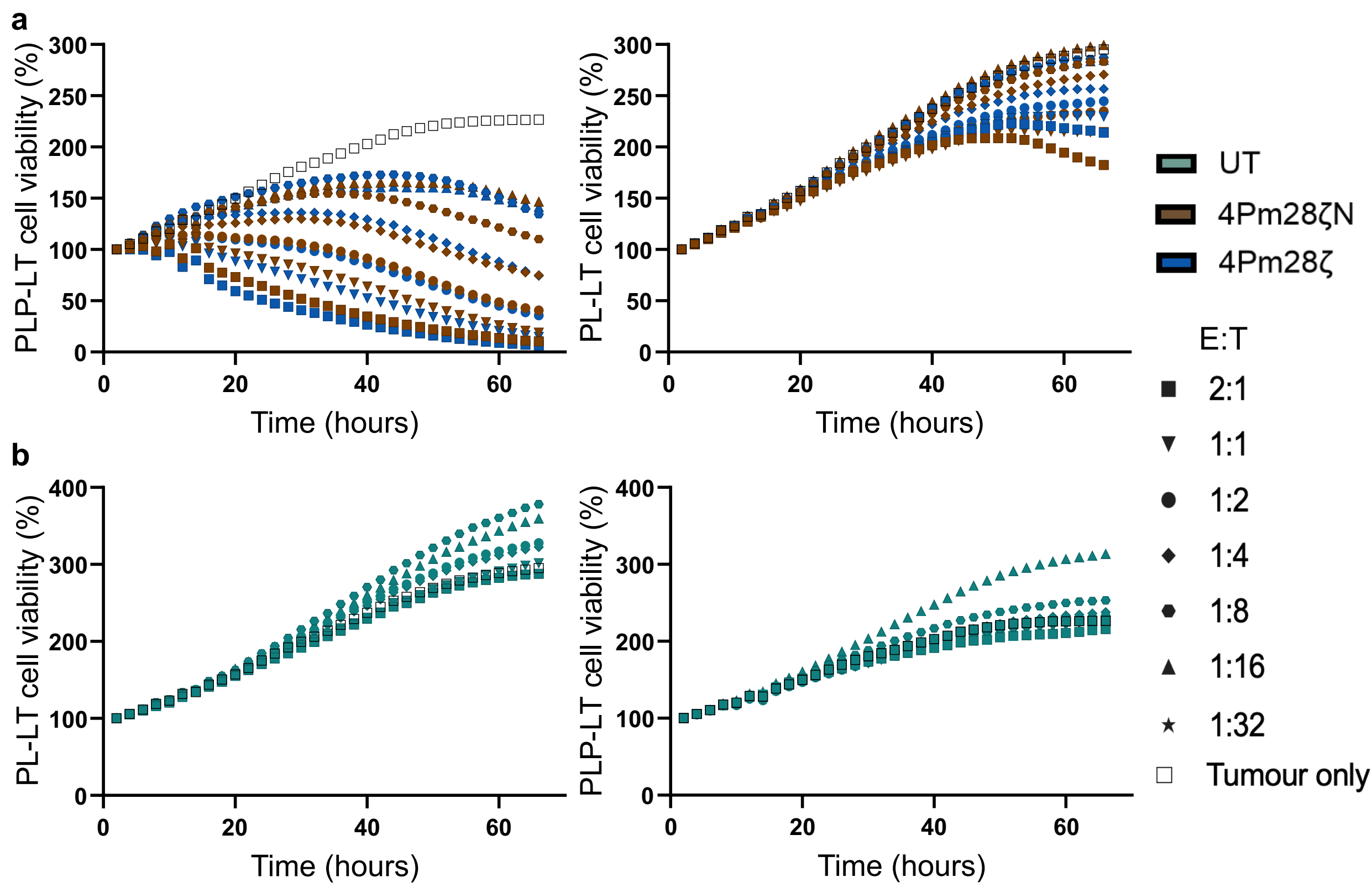

a
PLP-LT cell viability (%)
PL-LT cell viability (%)
Time (hours)
b
PL-LT cell viability (%)
PLP-LT cell viability (%)
Time (hours)
UT
4Pm28ζN
4Pm28ζ
E:T
2:1
1:1
1:2
1:4
1:8
1:16
1:32
Tumour only
