# Supplementary material for: Dynamic SPECT imaging kinetic modelling characterises tracer influx and efflux in tumour-infiltrating CAR T cells
Source: EJNMMI Res. 2026 Aug 29;16:160. doi: 10.1186/s13550-026-01492-y (PMC13768539; doi:10.1186/s13550-026-01492-y)
Supplement: Supplementary file 4 [file 13550_2026_1492_MOESM4_ESM.pdf]

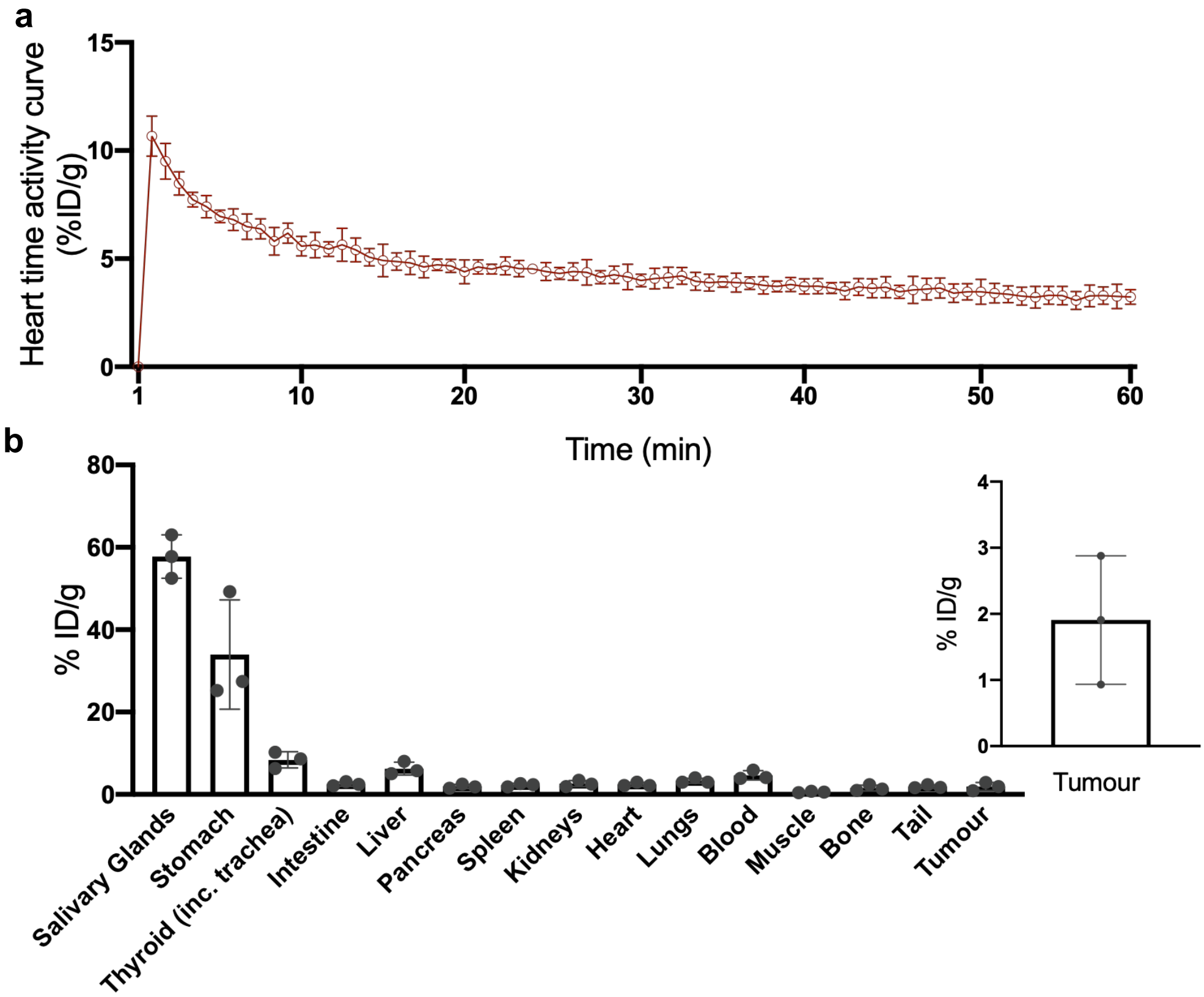
a
Heart time activity curve (%ID/g)
15
10
5
0
1
10
20
30
40
50
60
Time (min)
b
% ID/g
80
60
40
20
0
Salivary Glands
Stomach
Thyroid (inc. trachea)
Intestine
Liver
Pancreas
Spleen
Kidneys
Heart
Lungs
Blood
Muscle
Bone
Tail
Tumour
% ID/g
4
3
2
1
0
Tumour
