# Supplementary material for: Dynamic SPECT imaging kinetic modelling characterises tracer influx and efflux in tumour-infiltrating CAR T cells
Source: EJNMMI Res. 2026 Aug 29;16:160. doi: 10.1186/s13550-026-01492-y (PMC13768539; doi:10.1186/s13550-026-01492-y)
Supplement: Supplementary file 5 [file 13550_2026_1492_MOESM5_ESM.pdf]

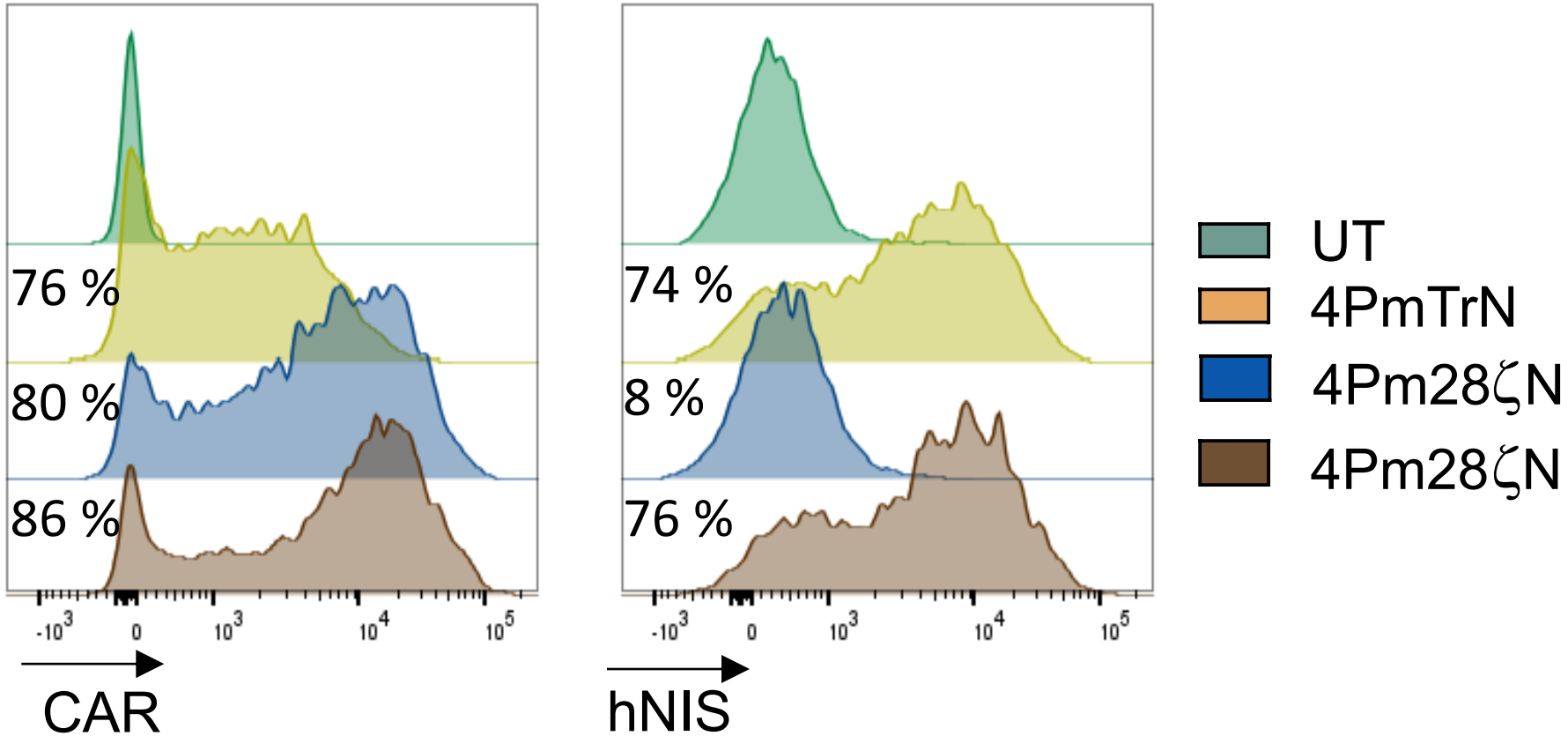

76 %
80 %
86 %
-10^3
0
10^3
10^4
10^5
CAR
74 %
8 %
76 %
-10^3
0
10^3
10^4
10^5
hNIS
UT
4PmTrN
4Pm28ζN
4Pm28ζN
