# Supplementary material for: Dynamic SPECT imaging kinetic modelling characterises tracer influx and efflux in tumour-infiltrating CAR T cells
Source: EJNMMI Res. 2026 Aug 29;16:160. doi: 10.1186/s13550-026-01492-y (PMC13768539; doi:10.1186/s13550-026-01492-y)
Supplement: Supplementary file 6 [file 13550_2026_1492_MOESM6_ESM.pdf]

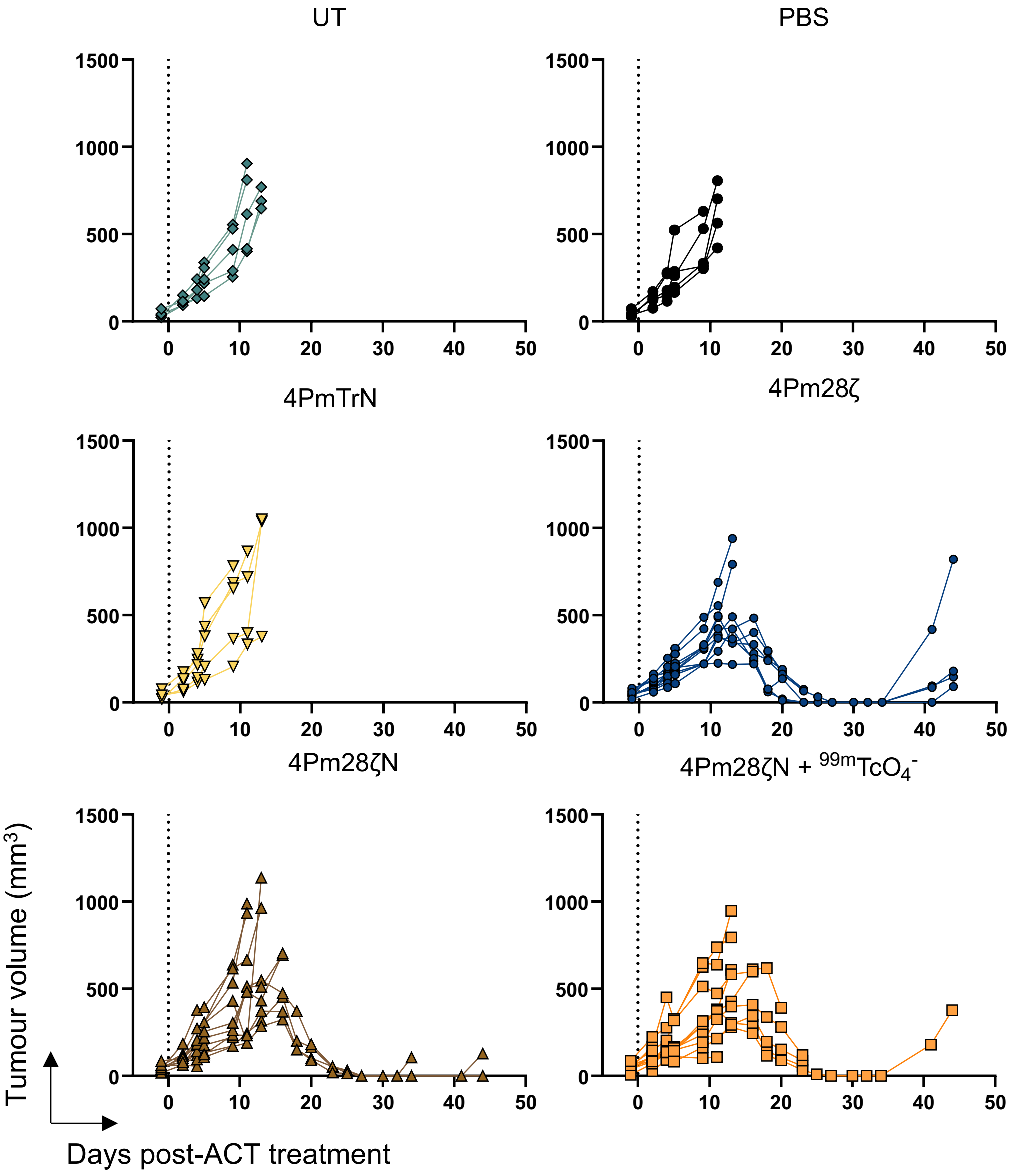
UT
PBS
4PmTrN
4Pm28ζ
4Pm28ζN
4Pm28ζN + 99mTcO4-
Tumour volume (mm3)
Days post-ACT treatment
0
500
1000
1500
0
10
20
30
40
50
