# Supplementary material for: Dynamic SPECT imaging kinetic modelling characterises tracer influx and efflux in tumour-infiltrating CAR T cells
Source: EJNMMI Res. 2026 Aug 29;16:160. doi: 10.1186/s13550-026-01492-y (PMC13768539; doi:10.1186/s13550-026-01492-y)
Supplement: Supplementary file 7 [file 13550_2026_1492_MOESM7_ESM.pdf]

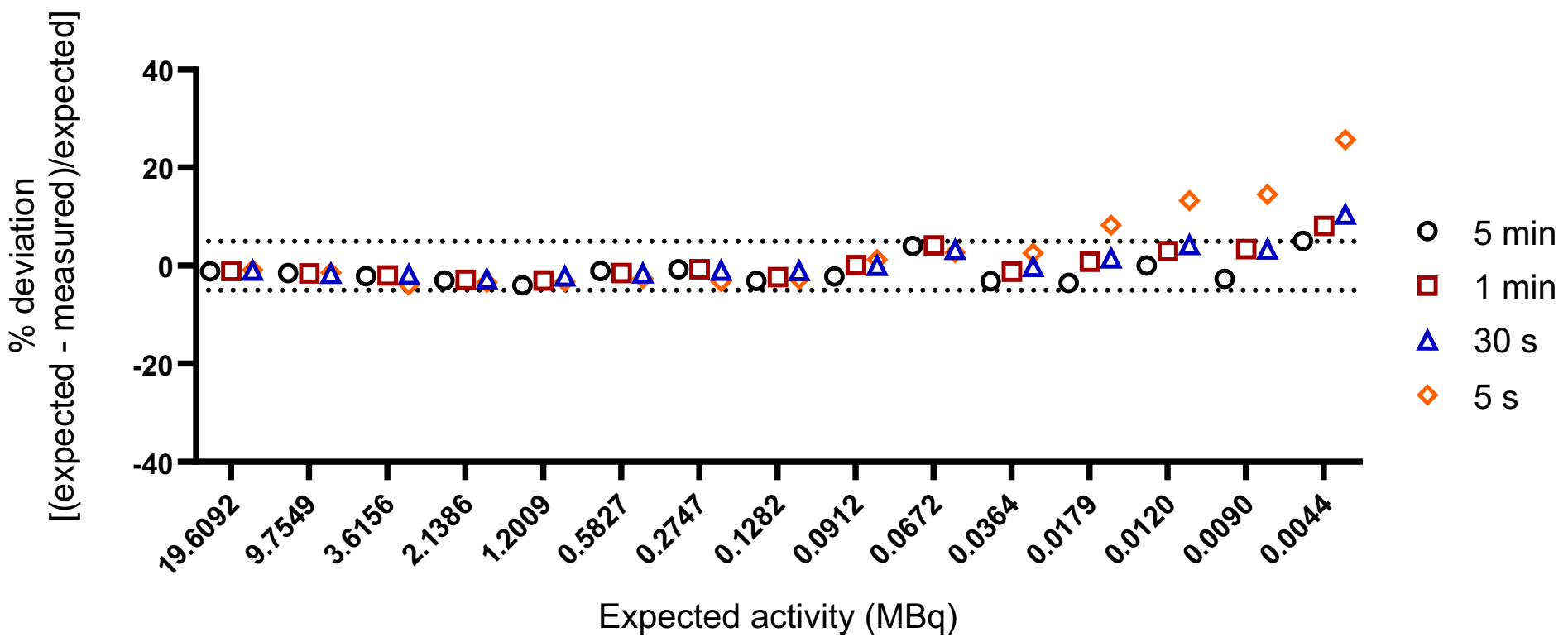
% deviation
[(expected - measured)/expected]
40
20
0
-20
-40
19.6092
9.7549
3.6156
2.1386
1.2009
0.5827
0.2747
0.1282
0.0912
0.0672
0.0364
0.0179
0.0120
0.0090
0.0044
Expected activity (MBq)
5 min
1 min
30 s
5 s
