# Supplementary material for: Dynamic SPECT imaging kinetic modelling characterises tracer influx and efflux in tumour-infiltrating CAR T cells
Source: EJNMMI Res. 2026 Aug 29;16:160. doi: 10.1186/s13550-026-01492-y (PMC13768539; doi:10.1186/s13550-026-01492-y)
Supplement: Supplementary file 8 [file 13550_2026_1492_MOESM8_ESM.docx]

Supplementary table

Table S1. Two-compartment kinetic model fitness

|  | | Value (min^-1^) | Standard Deviation | 95% confidence interval | Coefficient of variation (%) | Objective function | Scaled data variance |
| --- | --- | --- | --- | --- | --- | --- | --- |
| 4Pm28ζN (I) | K_1_ | 0.089 | 0.008 | 0.07-0.10 | 10.0 | 2.80 | 7.09 |
|  | K_2_ | 0.0363 | 0.009 | 0.01-0.05 | 27.1 |  |  |
| 4Pm28ζN (II) | K_1_ | 0.1604 | 0.017 | 0.12-0.19 | 10.5 | 2.91 | 6.26 |
|  | K_2_ | 0.0723 | 0.013 | 0.04-0.10 | 18.9 |  |  |
| 4Pm28ζN (III) | K_1_ | 0.1463 | 0.013 | 0.11-0.17 | 9.42 | 3.30 | 5.93 |
|  | K_2_ | 0.0440 | 0.009 | 0.02-0.06 | 22.1 |  |  |
| Group average | K_1_ | 0.14 | 0.014 | 0.10-0.17 | 10.3 | 3.09 | 0.065 |
|  | K_2_ | 0.056 | 0.012 | 0.03-0.08 | 21.6 |  |  |

|  | | Value (min^-1^) | Standard Deviation | 95% confidence interval | Coefficient of variation (%) | Objective function | Scaled data variance |
| --- | --- | --- | --- | --- | --- | --- | --- |
| 4Pm28ζ (I) | K_1_ | 0.0084 | 0.003 | 0.002-0.014 | 36.1 | 0.80 | 49.4 |
|  | K_2_ | 0.099 | 0.005 | -0.018-0.21 | 56.4 |  |  |
| 4Pm28ζ (II) | K_1_ | 0.0774 | 0.016 | 0.04-0.11 | 21.2 | 0.072 | 12.4 |
|  | K_2_ | 0.257 | 0.062 | 0.126-0.388 | 24.1 |  |  |
| 4Pm28ζ (III) | K_1_ | 0.196 | 0.005 | 0.091-0.30 | 25.2 | 0.99 | 7.67 |
|  | K_2_ | 0.448 | 0.012 | 0.19-0.706 | 27.2 |  |  |
| Group average | K_1_ | 0.07 | 0.014 | 0.04-0.10 | 19.0 | 0.5 | 0.086 |
|  | K_2_ | 0.21 | 0.051 | 0.11-0.32 | 23.4 |  |  |

Table S1. SAAM II-derived metrics for the two-compartment kinetic model applied to dynamic SPECT data from 4Pm28ζN-treated (top) and 4Pm28ζ control (bottom) tumours. For each estimated kinetic parameter, the table reports the fitted value (min-1), standard deviation, confidence interval and coefficient of variation, together with the objective function value and scaled data variance as global indicators of fit quality. Datasets (I), (II), (III) correspond to individual animals.
